# Supplementary material for: DNA methylation at retrotransposons protects the germline by preventing NRF1-mediated activation
Source: EMBO Rep. 2025 Aug 4;26(17):4312–39. doi: 10.1038/s44319-025-00526-1 (PMC12420836; doi:10.1038/s44319-025-00526-1)
Supplement: Supplementary file 1 — Appendix [file 44319_2025_526_MOESM1_ESM.pdf]

APPENDIX FIGURES

Table of content:

Appendix Figure S1……………………………………………………………………..…...1
Appendix Figure S2……………………………………………………………………..…...3
Appendix Figure S3……………………………………………………………………..…...5
Appendix Figure S4……………………………………………………………………..…...7
Appendix Figure S5……………………………………………………………………..…...9
Appendix Figure S6……………………………………………………………………..…..11
Appendix Figure S7……………………………………………………………………..…..13
Appendix Figure S8……………………………………………………………………..…..15

Appendix Figure S1

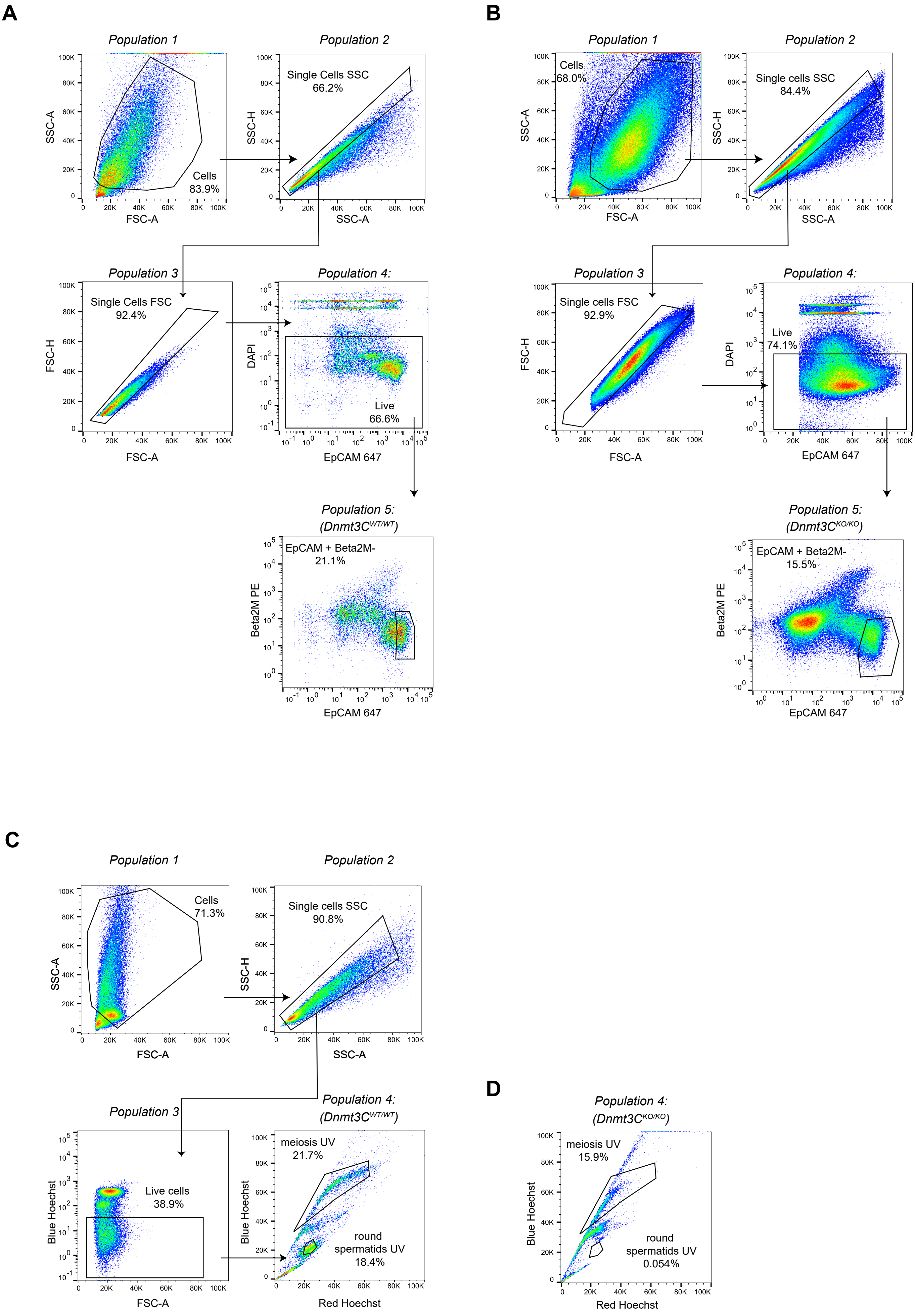

Appendix Fig. S1: Related to Figure1

(A) Gating strategy used for *Dnmt3C*$^{WT/WT}$ Spermatogonia (Spg) sorting. In the first three gates, healthy singlet cells are sorted. The fourth gate excludes dead cells that are DAPI-positive. The final gating strategy sorts cells, which are β2-Microglobulin(Beta2M)-PE negative and EpCAM-AF647 positive. **(B)** Same gating strategy as in (A) for *Dnmt3C*$^{KO/KO}$ Spg. **(C)** and **(D)** Gating strategy used for Spermatocyte (Spc) sorting. Population 1 and 2 (C) sort singlet healthy cells. The third population of (C) excludes dead cells stained by Propidium Iodide. The fourth population sorts Spc dependent on their DNA content and cell size stained by Hoechst3342 in (C) for wild-type and (D) for *Dnmt3C*$^{KO/KO}$ Spc, where not all meiotic stages are present and round spermatids are absent..

Appendix Figure S2

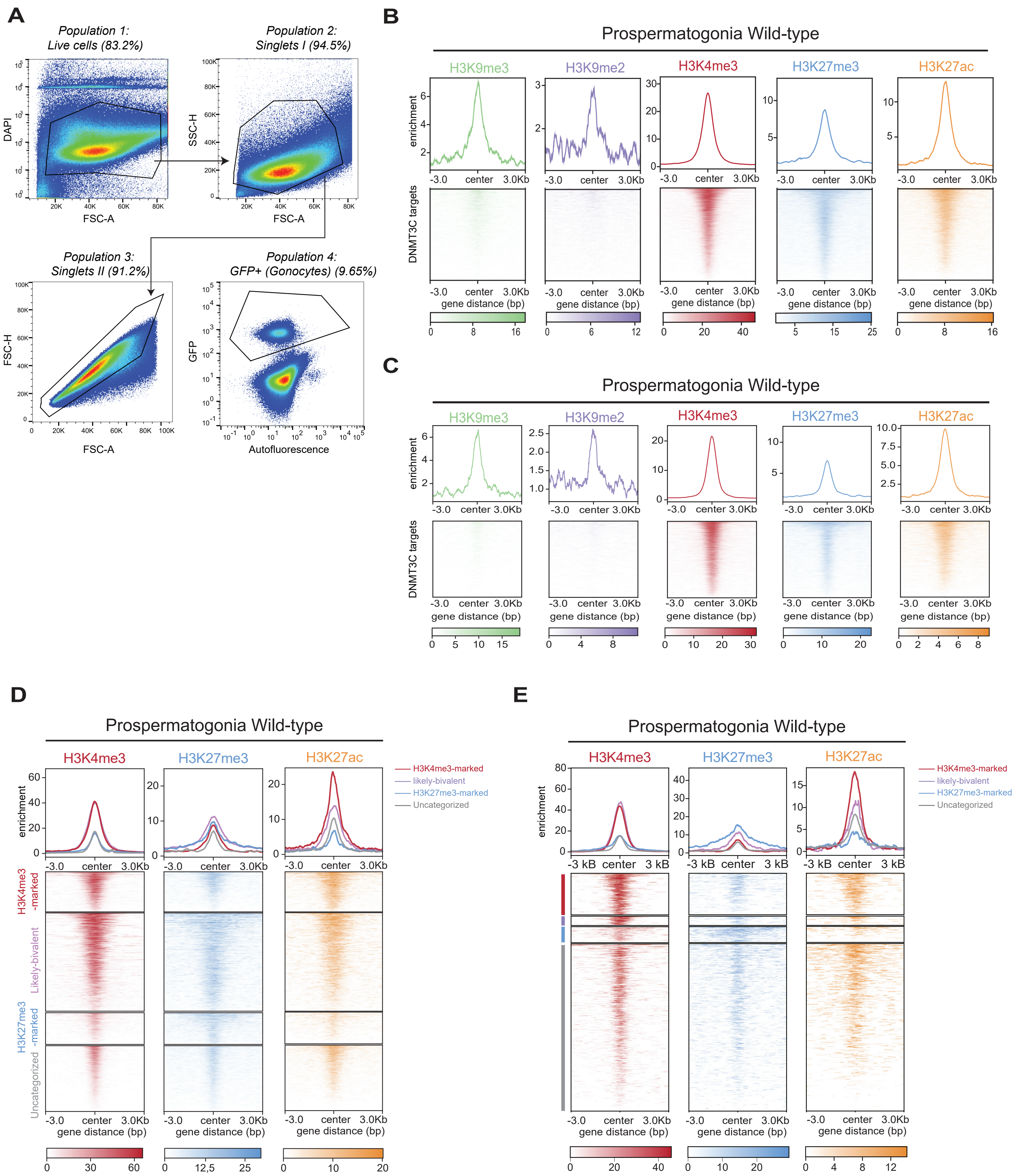

Appendix Fig. S2: Related to Figure2

(A) Gating strategy used for Prospermatogonia (ProSpg) sorting. In the first gate, live cells that are DAPI-negative are sorted. Next, singlets are sorted by side (population 2) and forward scattering (population 3). In the final gating GFP-positive cells are sorted. (A) Gating strategy used for Prospermatogonia (ProSpg) sorting. In the first gate, live cells that are DAPI-negative are sorted. Next, singlets are sorted by side (population 2) and forward scattering (population 3). In the final gating GFP-positive cells are sorted. **(B)** Heatmaps displaying normalized coverage and metaplots showing mean enrichment of H3K4me3, H3K27me3, H3K9me2, H3K9me3 and H3K27ac CUT&Tag, centered on DNMT3C targets in sorted *Dnmt3C*$^{WT/WT}$ ProSpg. **(C)** as in (B), showing data from an individual biological replicate. **(D)** Heatmaps showing normalized coverage and metaplots displaying mean enrichment of H3K4me3, H3K27me3 and H3K27ac CUT&Tag centered on likely-bivalent, H3K4me3-marked, H3K27me3-marked and uncategorized DNMT3C-targets in sorted ProSpg from two biological replicates. **(E)** as in (D), showing data from an individual biological replicate.

Appendix Figure S3

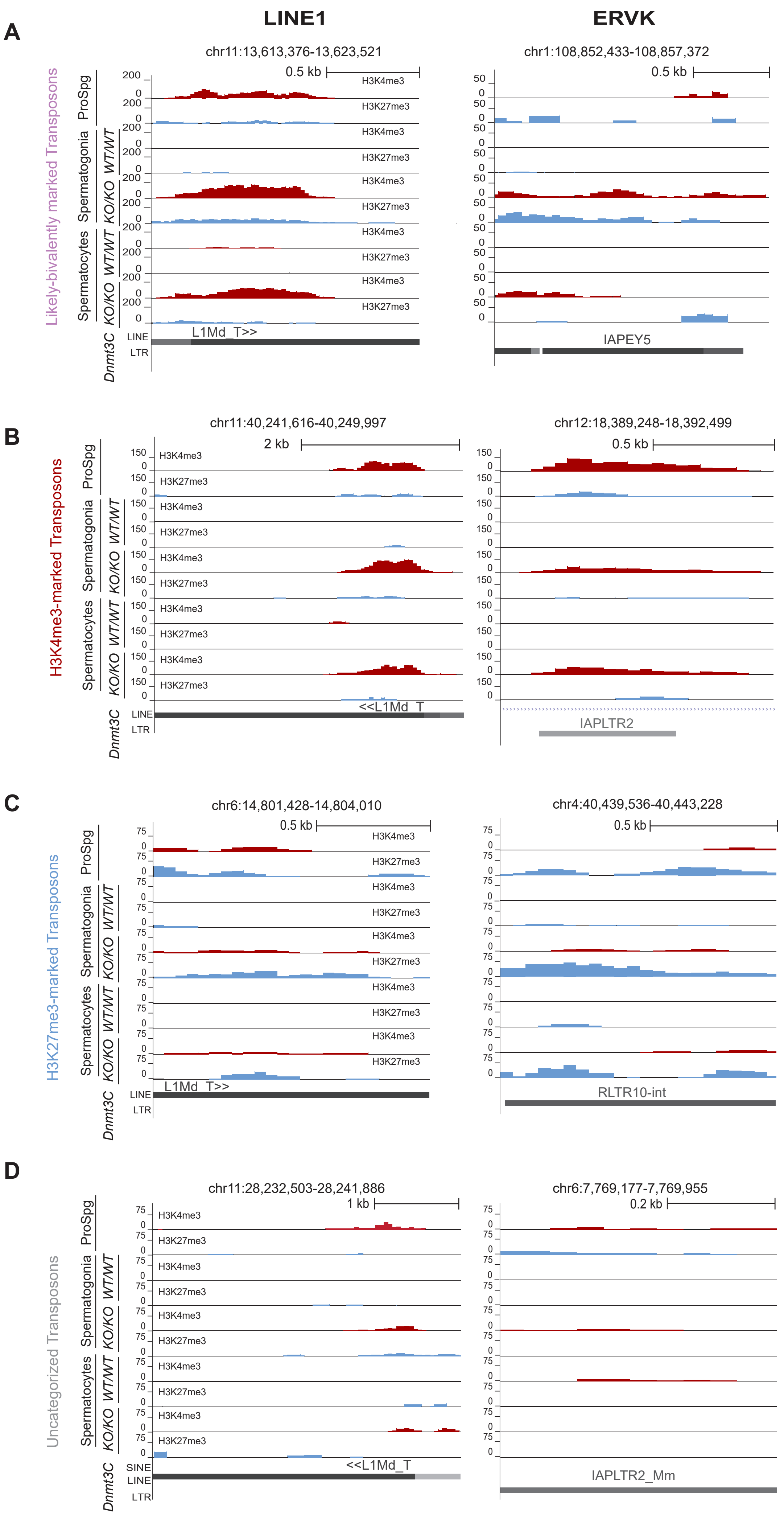

Appendix Fig. S3: Related to Figure2

Representative track examples of H3K4me3 and H3K27me3 CUT&Tag LINE1 and ERVK copies classified into likely-bivalent **(A)**, H3K4me3-marked **(B)**, H3K27me3-marked **(C)** and Uncategorized categories **(D)** from ProSpg as well as *Dnmt3C*$^{WT/WT}$ and *Dnmt3C*$^{KO/KO}$ Spg and Spc.

Appendix Figure S4

A

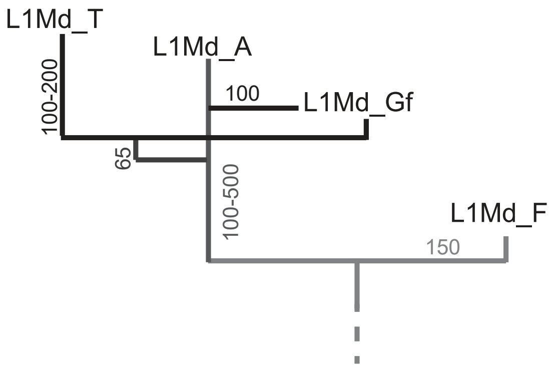

B

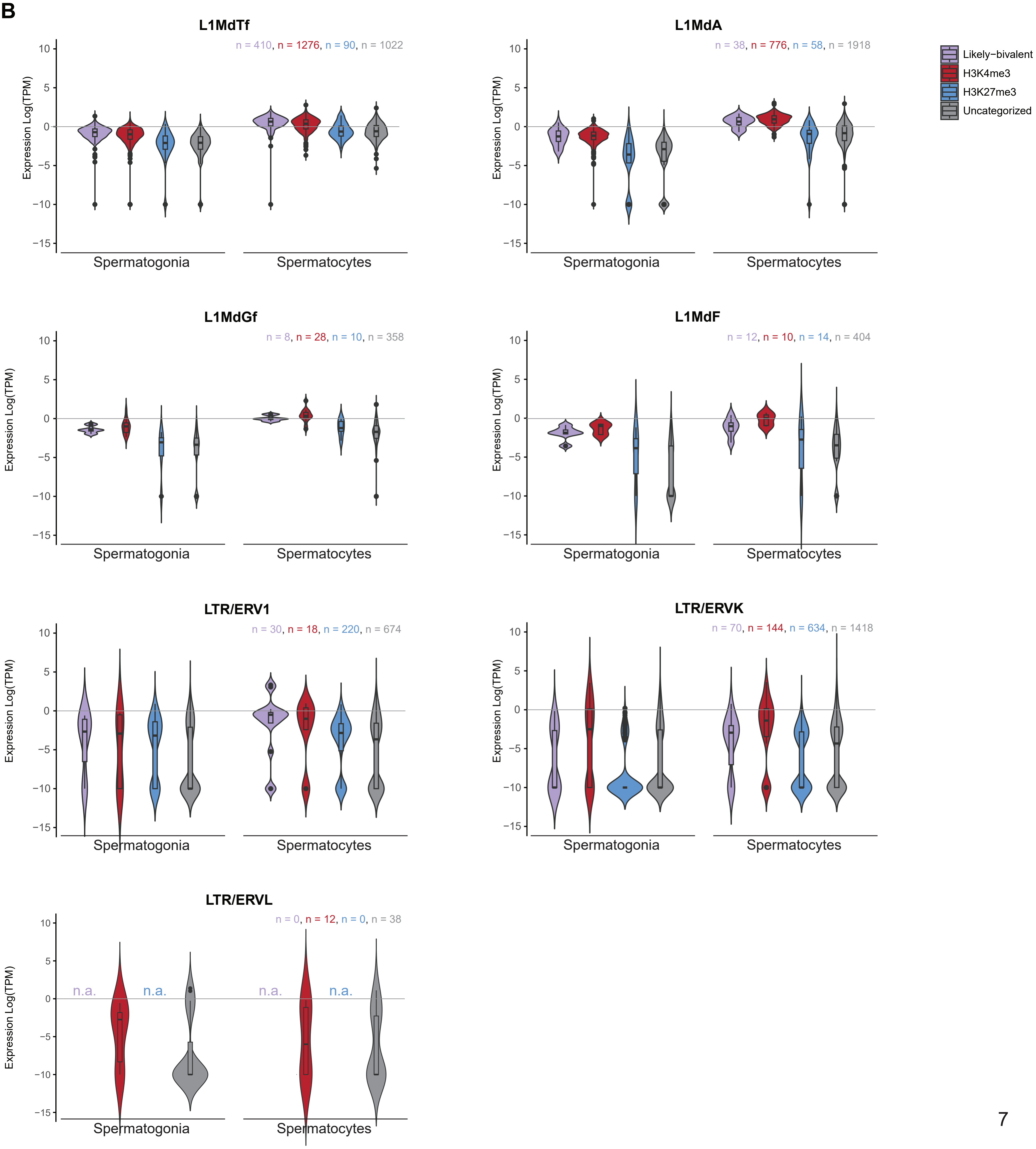

Appendix Fig. S4: Related to Figure2

(A) Phylogenetic tree illustrating the evolutionary age of L1 elements. This image is modified from (Sookdeo et al. 2013). **(B)** Violin plots showing log(TPM) expression values obtained from uniquely mapped reads of three biological replicates using TElocal of the TE subfamilies L1MdT, L1MDA, L1MdG, L1MdF, ERVL, ERV1, ERVK categorized into likely-bivalent, H3K4me3-marked, H3K27me3-marked and Uncategorized as in Appendix Fig. S3.

Appendix Figure S5

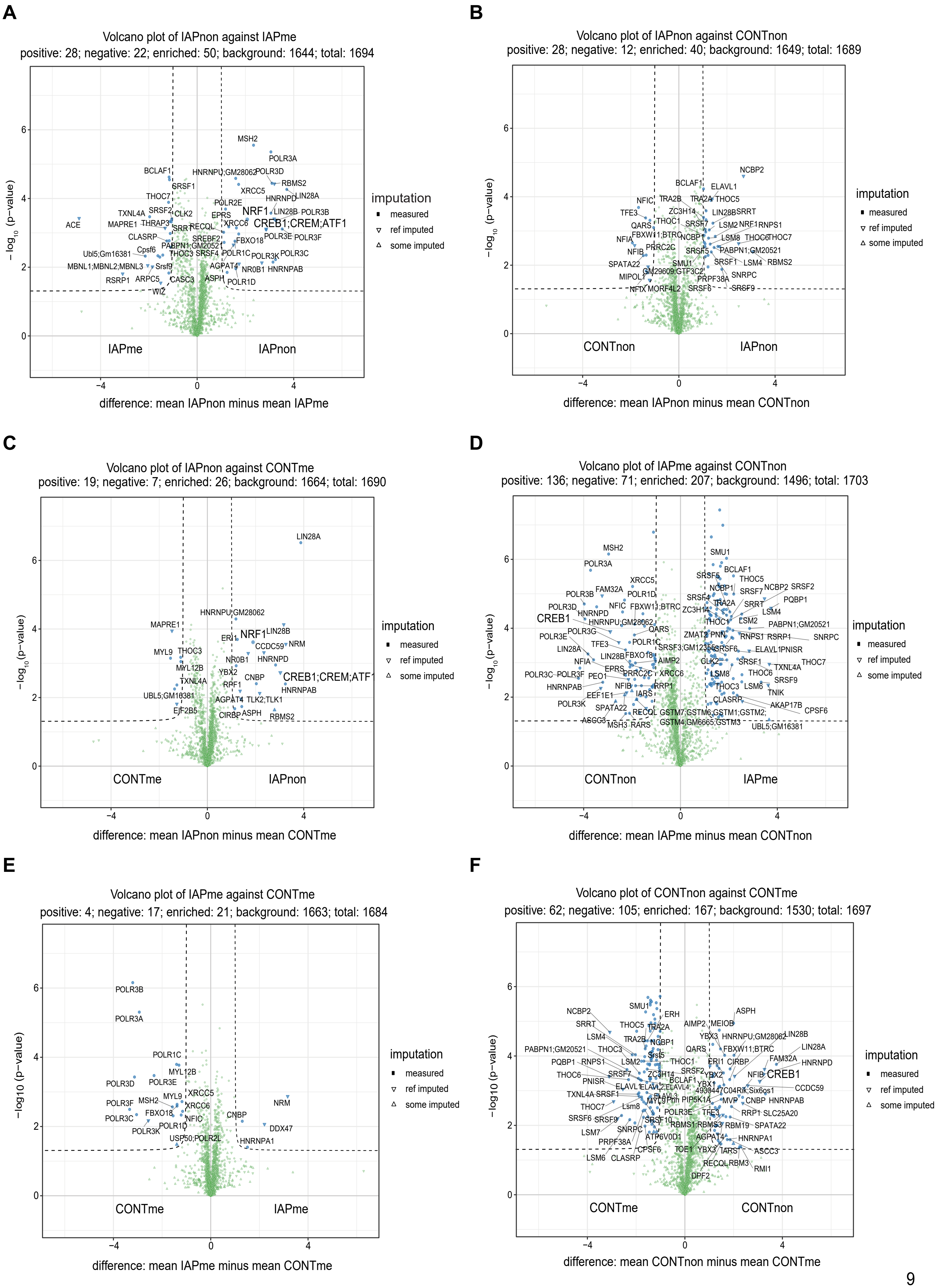

Appendix Fig. S5: Related to Figure3

Volcano plots showing the difference in mean values and $-\log_{10}$p-values of the original data on three biological replicates from LC-MS data, illustrating protein enrichment across bait probes. Comparisons include **(A)** unmethylated (non) IAP vs. methylated (me) IAP, **(B)** IAPnon vs. unmethylated scrambled control (CONTnon), **(C)** IAPnon vs. CONTme, **(D)** IAPme vs. CONTnon, **(E)** IAPme vs. CONTme, and **(F)** CONTnon vs. CONTme.

Appendix Figure S6

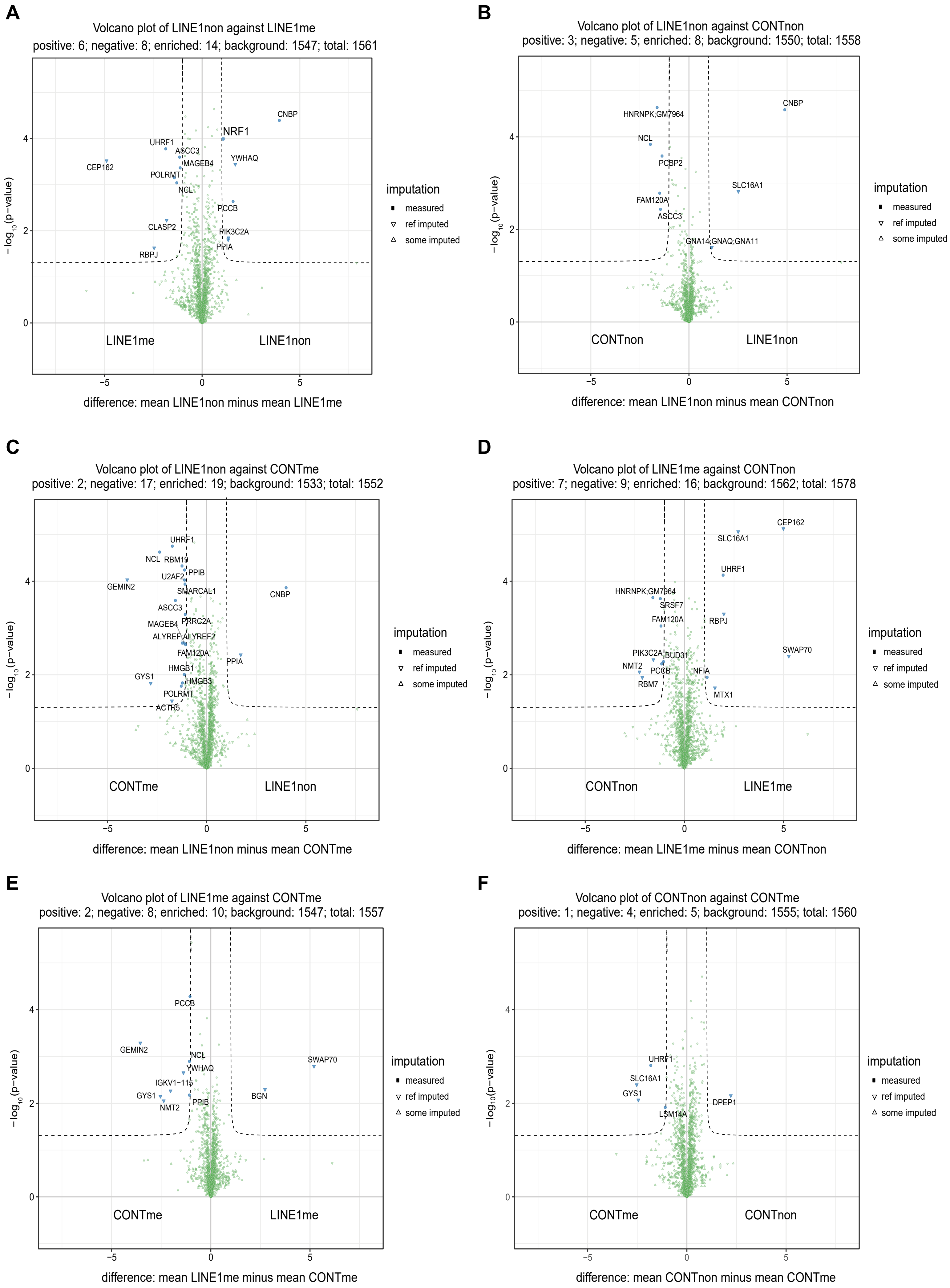

Appendix Fig. S6: Related to Figure3

Volcano plots showing the difference in mean values and -$\log_{10}$p-values of the original data on three biological replicates from LC-MS data, illustrating protein enrichment across bait probes. As in Appendix Fig. S5, comparisons include **(A)** LINE1non vs. LINE1me, **(B)** LINE1non vs. CONTnon, **(C)** LINE1non vs. CONTme, **(D)** LINE1me vs. CONTnon, **(E)** LINE1me vs. CONTme, **(F)** CONTnon vs. CONTme.

Appendix Figure S7

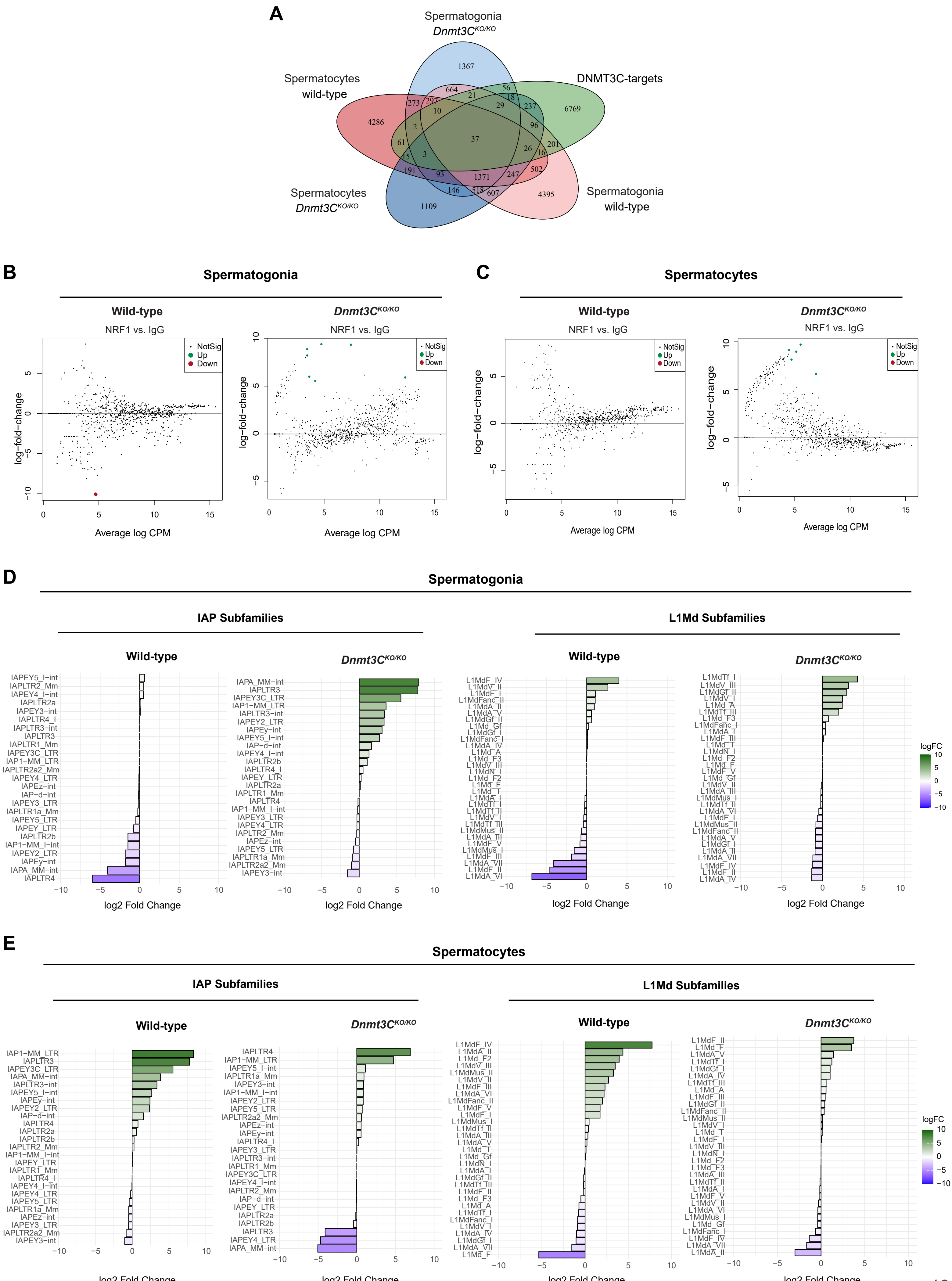

Appendix Fig. S7: Related to Figure4

(A) Venn Diagram showing overlaps between NRF1 CUT&Tag peaks in wild-type and *Dnmt3C*$^{KO/KO}$ Spg and Spc with DNMT3C targets. **(B)** MA plots showing differential NRF1 enrichment relative to IgG, in wild-type and *Dnmt3C*$^{KO/KO}$ spermatogonia. TE read counts were quantified using RepEnrich. Significantly upregulated (Up,red), downregulated (Down, blue) and Non-significant (NotSig, black) DNMT3C targets identified using edgeR. Statistics were conducted using two biological replicates **(C)** as in (B) for spermatocytes. **(D)** Bar plots showing log2FC NRF1 enrichment over IgG enrichment for differentially enriched IAP (left) and L1 (right) subfamilies in wild-type and *Dnmt3C*$^{KO/KO}$ spermatogonia. **(E)** as in (D) for spermatocytes. Venn Diagram showing overlaps between NRF1 CUT&Tag peaks in wild-type and *Dnmt3C*$^{KO/KO}$ Spg and Spc with DNMT3C targets.

Appendix Figure S8

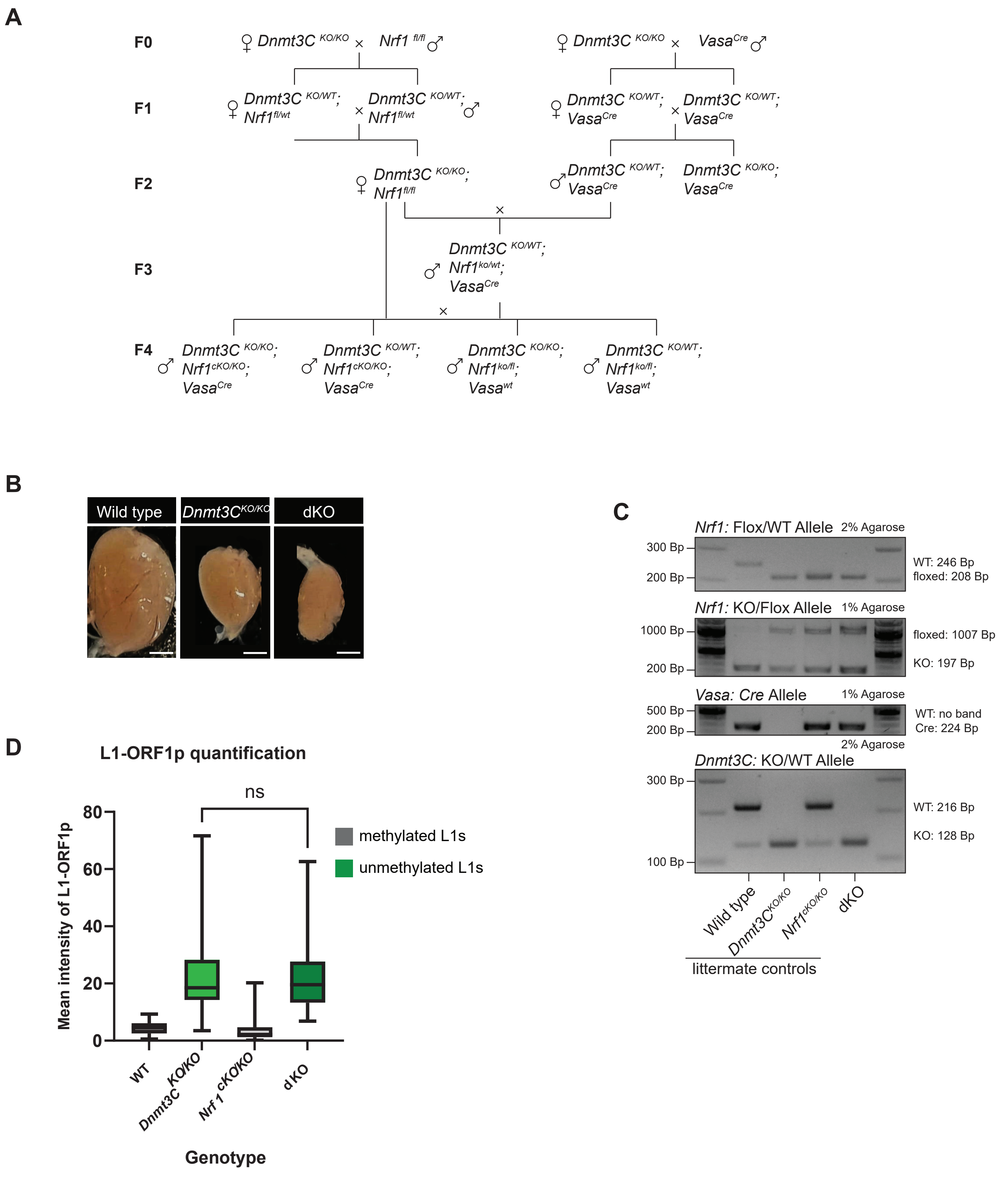

Appendix Fig. S8: Related to Figure5

(A) Breeding scheme to obtain dKO (*Dnmt3C$^{KO/KO}$*, *Nrf1$^{cKO/KO}$*) and littermate controls. Crosses (x) are indicated of four different generations (F). **(B)** Representative image of testes from *Dnmt3C$^{KO/KO}$* and dKO of 5-week-old mice showing hypogonadism phenotype. Scale bar 2mm. **(C)** Representative agarose gels showing genotyping results for *Vasa-Cre*, *Dnmt3C*, and *Nrf1* floxed (Flox) and KO alleles in wild-type, *Dnmt3C$^{KO/KO}$*, *Nrf1$^{cKO/KO}$* and dKO mutants. The percentage of agarose is indicated above each gel and the expected band size next to each gel. **(D)** L1-ORF1p signal quantification from panel Figure 5E; cells counted from one biological replicate: WT, n=89; Dnmt3CKO/KO , n= 79; Nrf1cKO/KO, n=85; dKO, n= 118; Methylated L1s are depicted in gray and unmethylated L1s in green. Statistical analysis (t-test), error bars (s.d) and graph generation were carried out using GraphPad Prism version 10.
