# Supplementary figures and images for: DNA methylation at retrotransposons protects the germline by preventing NRF1-mediated activation (part 1 of 2)
Source: EMBO Rep. 2025 Aug 4;26(17):4312–39. doi: 10.1038/s44319-025-00526-1 (PMC12420836; doi:10.1038/s44319-025-00526-1)

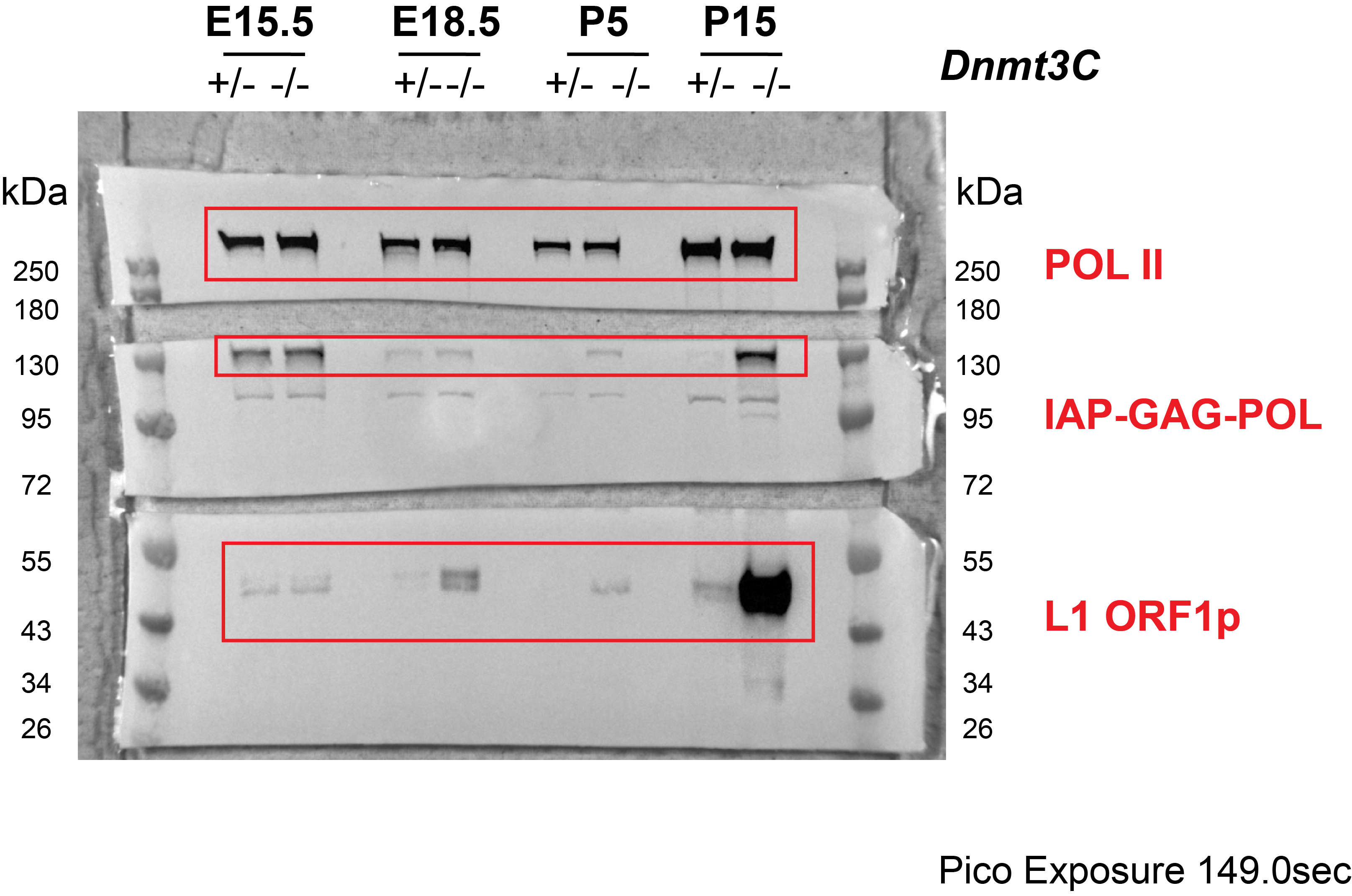

Supplement: Supplementary file 7 — Source data Fig. 1 [file 44319_2025_526_MOESM7_ESM.zip › Figure 1/1B/western_IAP-GAG-POL_L1ORF1p.png]

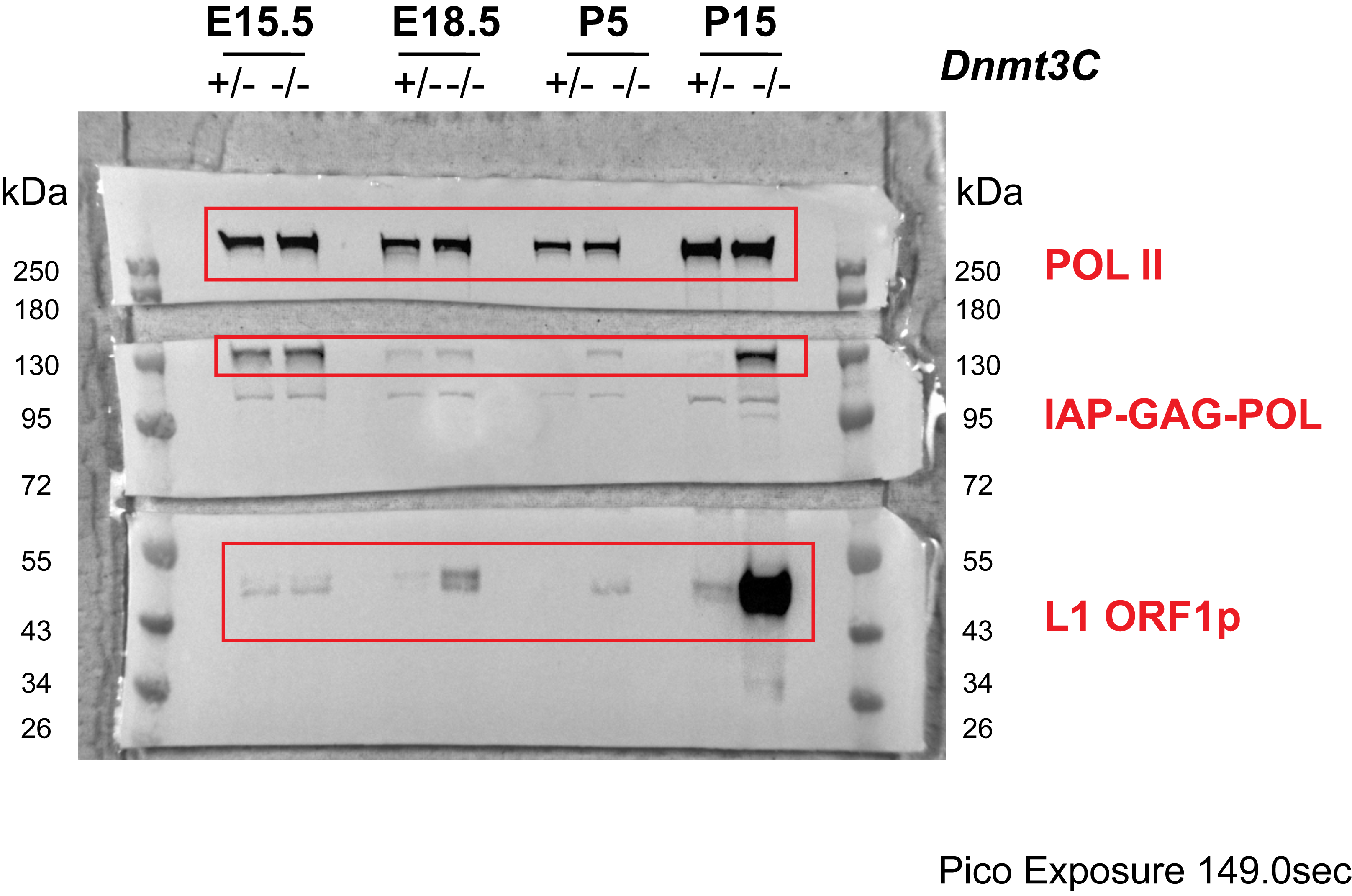

Supplement: Supplementary file 7 — Source data Fig. 1 [file 44319_2025_526_MOESM7_ESM.zip › Figure 1/1B/Western_IAP-GAG-POL_L1ORF1p_uncropped.tif]

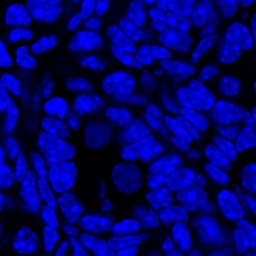

Supplement: Supplementary file 7 — Source data Fig. 1 [file 44319_2025_526_MOESM7_ESM.zip › Figure 1/1A/E18.5_WT/DAPI_E18.5_WT_ Series009.jpg]

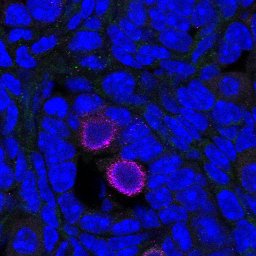

Supplement: Supplementary file 7 — Source data Fig. 1 [file 44319_2025_526_MOESM7_ESM.zip › Figure 1/1A/E18.5_WT/MERGE-E18.5_WT_ Series009.jpg]

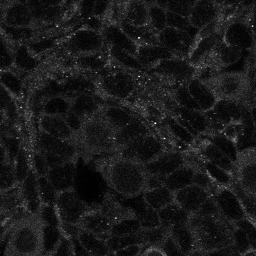

Supplement: Supplementary file 7 — Source data Fig. 1 [file 44319_2025_526_MOESM7_ESM.zip › Figure 1/1A/E18.5_WT/merge_E18.5_WT_Series009.tif]

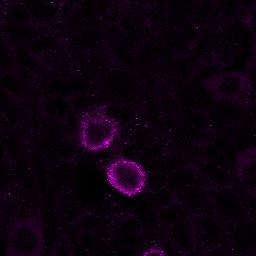

Supplement: Supplementary file 7 — Source data Fig. 1 [file 44319_2025_526_MOESM7_ESM.zip › Figure 1/1A/E18.5_WT/TRA98_E18.5_WT_ Series009.jpg]

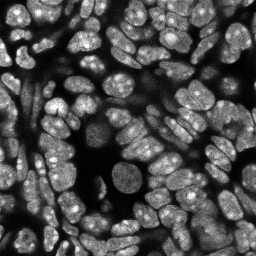

Supplement: Supplementary file 7 — Source data Fig. 1 [file 44319_2025_526_MOESM7_ESM.zip › Figure 1/1A/E18.5_WT/E18.5_WT_all_channels_Series009.tif]

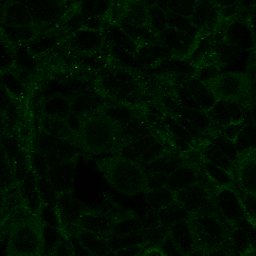

Supplement: Supplementary file 7 — Source data Fig. 1 [file 44319_2025_526_MOESM7_ESM.zip › Figure 1/1A/E18.5_WT/L1ORF1_E18.5_WT_Series009.jpg]

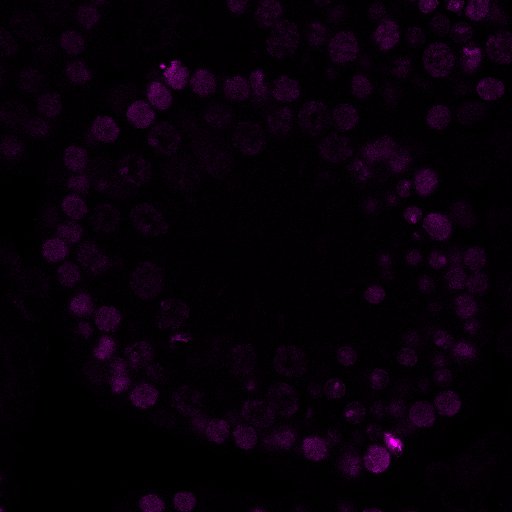

Supplement: Supplementary file 7 — Source data Fig. 1 [file 44319_2025_526_MOESM7_ESM.zip › Figure 1/1A/P30_WT/TRA98_P30_WT_Series011.jpg]

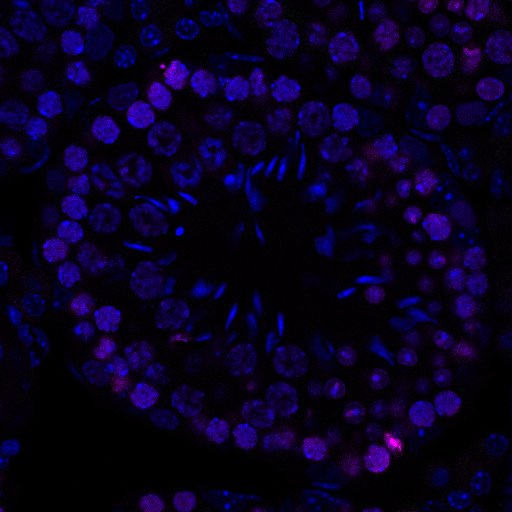

Supplement: Supplementary file 7 — Source data Fig. 1 [file 44319_2025_526_MOESM7_ESM.zip › Figure 1/1A/P30_WT/Merge_P30_WT_Series011.jpg]

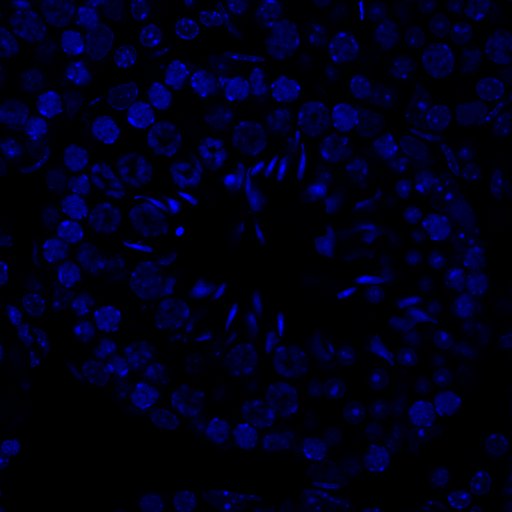

Supplement: Supplementary file 7 — Source data Fig. 1 [file 44319_2025_526_MOESM7_ESM.zip › Figure 1/1A/P30_WT/DAPI_P30_WT_Series011.jpg]

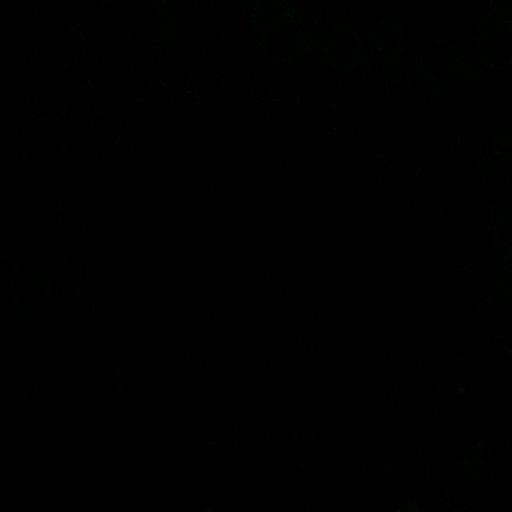

Supplement: Supplementary file 7 — Source data Fig. 1 [file 44319_2025_526_MOESM7_ESM.zip › Figure 1/1A/P30_WT/L1ORF1_P30_WT_Series011.jpg]

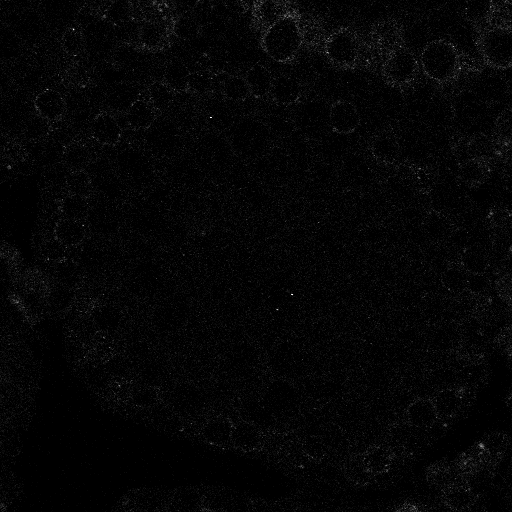

Supplement: Supplementary file 7 — Source data Fig. 1 [file 44319_2025_526_MOESM7_ESM.zip › Figure 1/1A/P30_WT/p30_WT_Series011_merge.tif]

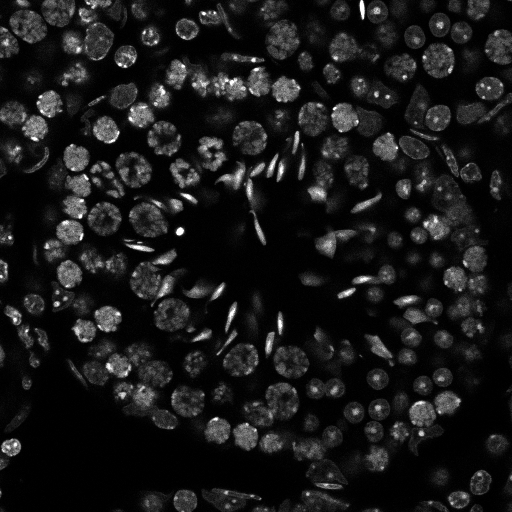

Supplement: Supplementary file 7 — Source data Fig. 1 [file 44319_2025_526_MOESM7_ESM.zip › Figure 1/1A/P30_WT/p30_WT_Series011_all_channels.tif]

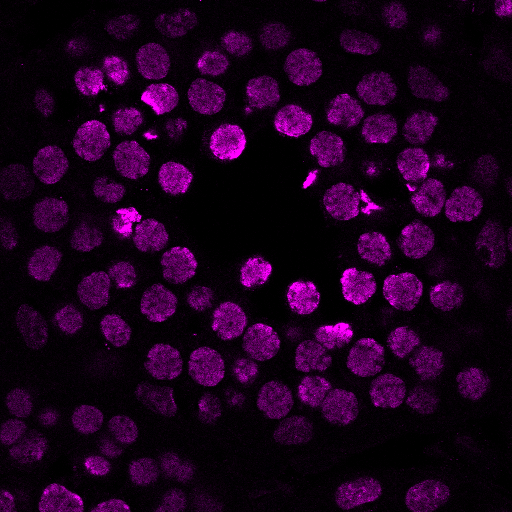

Supplement: Supplementary file 7 — Source data Fig. 1 [file 44319_2025_526_MOESM7_ESM.zip › Figure 1/1A/P20_WT/TRA98_P20_WT_Series001.png]

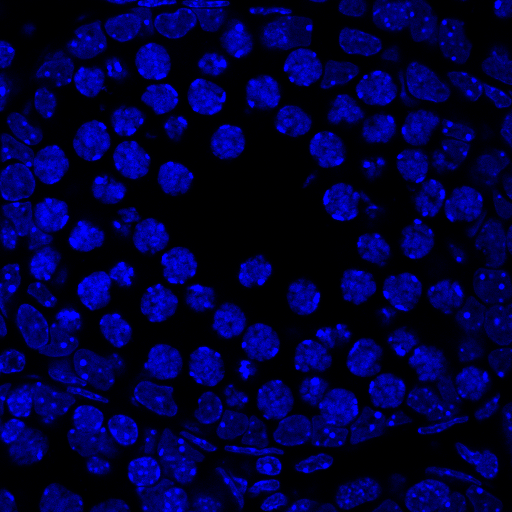

Supplement: Supplementary file 7 — Source data Fig. 1 [file 44319_2025_526_MOESM7_ESM.zip › Figure 1/1A/P20_WT/DAPI_P20_WT_Series001.png]

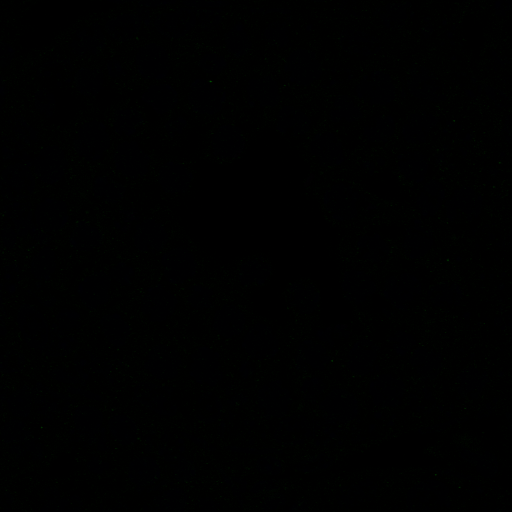

Supplement: Supplementary file 7 — Source data Fig. 1 [file 44319_2025_526_MOESM7_ESM.zip › Figure 1/1A/P20_WT/L1ORF1_P20_WT_Series001.png]

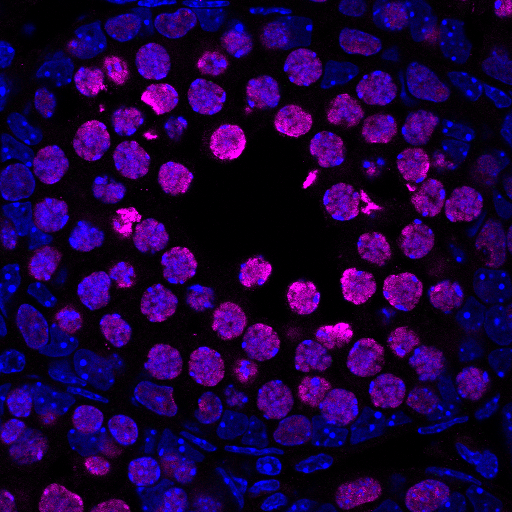

Supplement: Supplementary file 7 — Source data Fig. 1 [file 44319_2025_526_MOESM7_ESM.zip › Figure 1/1A/P20_WT/merge_P20_WT_Series001.png]

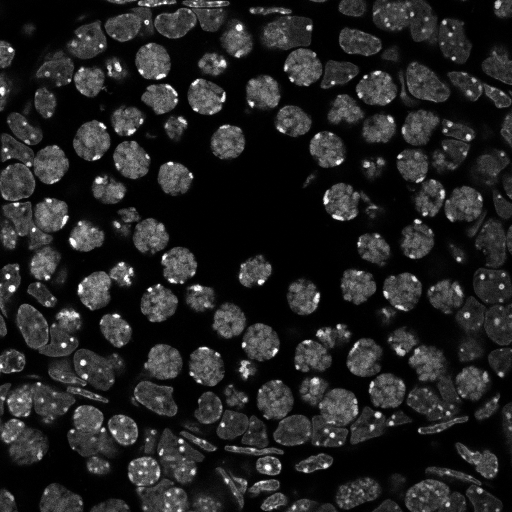

Supplement: Supplementary file 7 — Source data Fig. 1 [file 44319_2025_526_MOESM7_ESM.zip › Figure 1/1A/P20_WT/p20_WT_Series001_all_channels.tif]

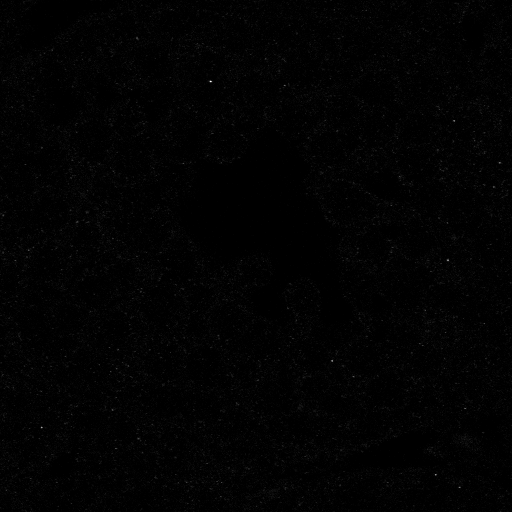

Supplement: Supplementary file 7 — Source data Fig. 1 [file 44319_2025_526_MOESM7_ESM.zip › Figure 1/1A/P20_WT/p20_WT_Series001_merge.tif]

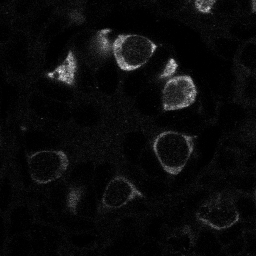

Supplement: Supplementary file 7 — Source data Fig. 1 [file 44319_2025_526_MOESM7_ESM.zip › Figure 1/1A/P5_KO/P5_3CKO_Series001_merge.tif]

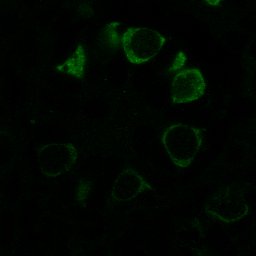

Supplement: Supplementary file 7 — Source data Fig. 1 [file 44319_2025_526_MOESM7_ESM.zip › Figure 1/1A/P5_KO/L1ORF1_P5_3CKO_Series001.jpg]

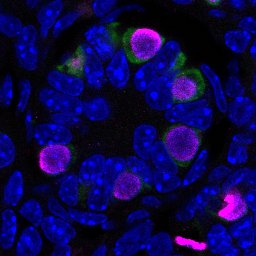

Supplement: Supplementary file 7 — Source data Fig. 1 [file 44319_2025_526_MOESM7_ESM.zip › Figure 1/1A/P5_KO/Merge_P5_3CKO_Series001.jpg]

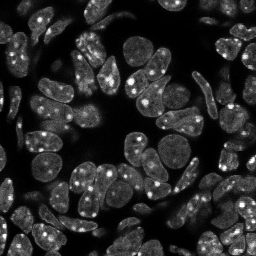

Supplement: Supplementary file 7 — Source data Fig. 1 [file 44319_2025_526_MOESM7_ESM.zip › Figure 1/1A/P5_KO/P5_3CKO_Series001_all_channels.tif]

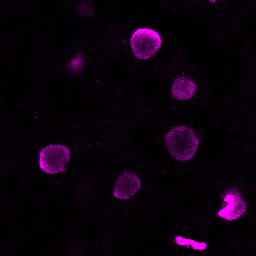

Supplement: Supplementary file 7 — Source data Fig. 1 [file 44319_2025_526_MOESM7_ESM.zip › Figure 1/1A/P5_KO/TRA98_P5_3CKO_Series001.jpg]

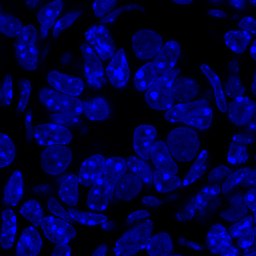

Supplement: Supplementary file 7 — Source data Fig. 1 [file 44319_2025_526_MOESM7_ESM.zip › Figure 1/1A/P5_KO/DAPI_P5_3CKO_ Series001.jpg]

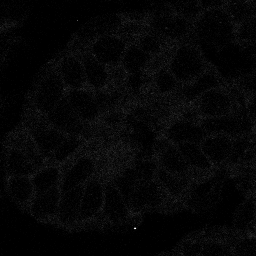

Supplement: Supplementary file 7 — Source data Fig. 1 [file 44319_2025_526_MOESM7_ESM.zip › Figure 1/1A/P10_WT/P10_WT_Series005_merge.tif]

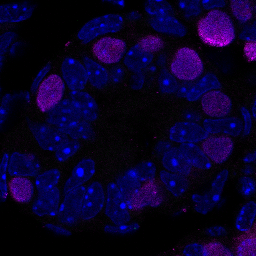

Supplement: Supplementary file 7 — Source data Fig. 1 [file 44319_2025_526_MOESM7_ESM.zip › Figure 1/1A/P10_WT/Merge_P10_WT_ Series005.png]

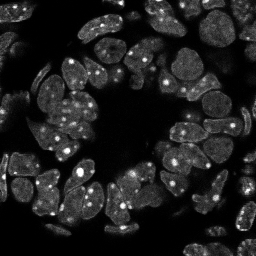

Supplement: Supplementary file 7 — Source data Fig. 1 [file 44319_2025_526_MOESM7_ESM.zip › Figure 1/1A/P10_WT/P10_WT_Series005_all_channels.tif]

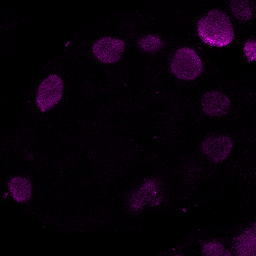

Supplement: Supplementary file 7 — Source data Fig. 1 [file 44319_2025_526_MOESM7_ESM.zip › Figure 1/1A/P10_WT/TRA98_P10_WT_Series005.png]

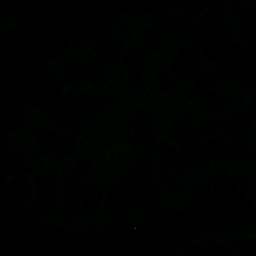

Supplement: Supplementary file 7 — Source data Fig. 1 [file 44319_2025_526_MOESM7_ESM.zip › Figure 1/1A/P10_WT/L1ORF1_P10_WT_Series005.png]

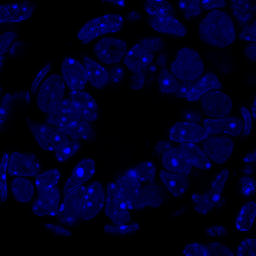

Supplement: Supplementary file 7 — Source data Fig. 1 [file 44319_2025_526_MOESM7_ESM.zip › Figure 1/1A/P10_WT/DAPI_P10_WT_ Series005.png]

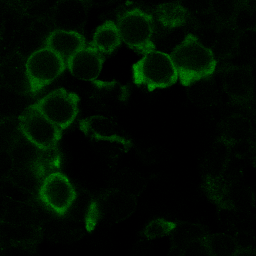

Supplement: Supplementary file 7 — Source data Fig. 1 [file 44319_2025_526_MOESM7_ESM.zip › Figure 1/1A/E15.5_WT/L1ORF1_E15.5_WT_Series004.png]

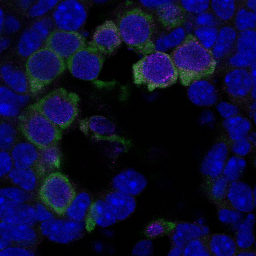

Supplement: Supplementary file 7 — Source data Fig. 1 [file 44319_2025_526_MOESM7_ESM.zip › Figure 1/1A/E15.5_WT/Merge_E15.5_WT_ Series004.png]

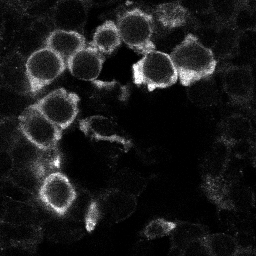

Supplement: Supplementary file 7 — Source data Fig. 1 [file 44319_2025_526_MOESM7_ESM.zip › Figure 1/1A/E15.5_WT/merge_E15.5_WT_Series004.tif]

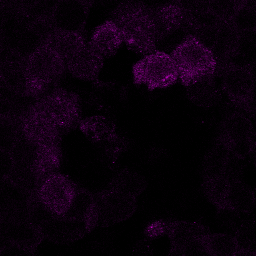

Supplement: Supplementary file 7 — Source data Fig. 1 [file 44319_2025_526_MOESM7_ESM.zip › Figure 1/1A/E15.5_WT/TRA98_E15.5_WT_Series004.png]

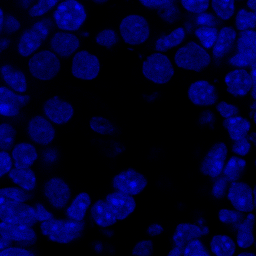

Supplement: Supplementary file 7 — Source data Fig. 1 [file 44319_2025_526_MOESM7_ESM.zip › Figure 1/1A/E15.5_WT/DAPI_E15.5_WT_ Series004.png]

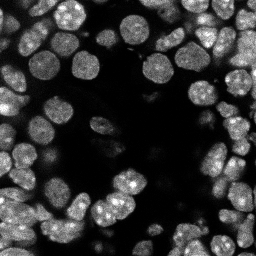

Supplement: Supplementary file 7 — Source data Fig. 1 [file 44319_2025_526_MOESM7_ESM.zip › Figure 1/1A/E15.5_WT/E15.5_WT_Series004_all_channels.tif]

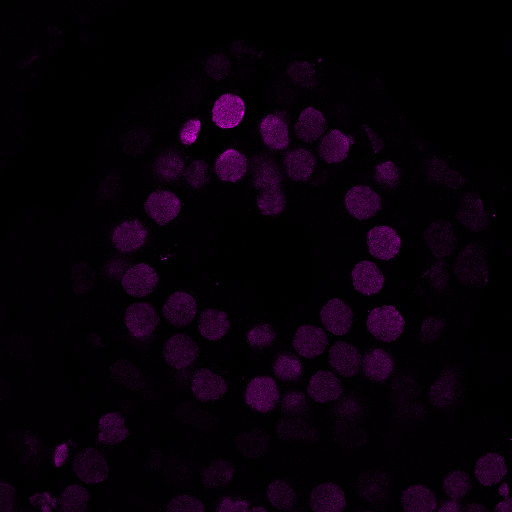

Supplement: Supplementary file 7 — Source data Fig. 1 [file 44319_2025_526_MOESM7_ESM.zip › Figure 1/1A/P15_WT/TRA98_P15_WT_Series003.png]

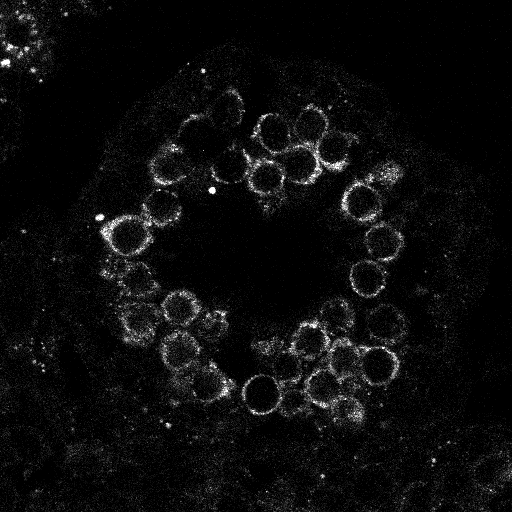

Supplement: Supplementary file 7 — Source data Fig. 1 [file 44319_2025_526_MOESM7_ESM.zip › Figure 1/1A/P15_WT/p15_D3C WT_Series003_merge.tif]

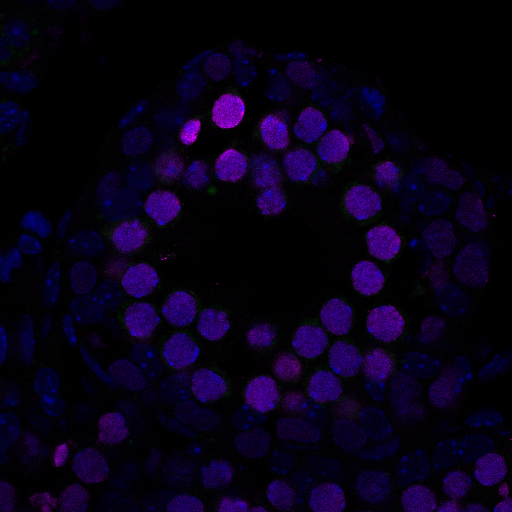

Supplement: Supplementary file 7 — Source data Fig. 1 [file 44319_2025_526_MOESM7_ESM.zip › Figure 1/1A/P15_WT/Merge_P15_WT_Series003.png]

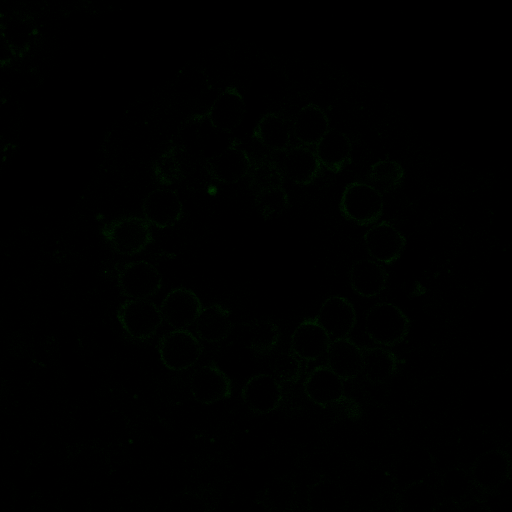

Supplement: Supplementary file 7 — Source data Fig. 1 [file 44319_2025_526_MOESM7_ESM.zip › Figure 1/1A/P15_WT/L1ORF1_P15_WT_Series003.png]

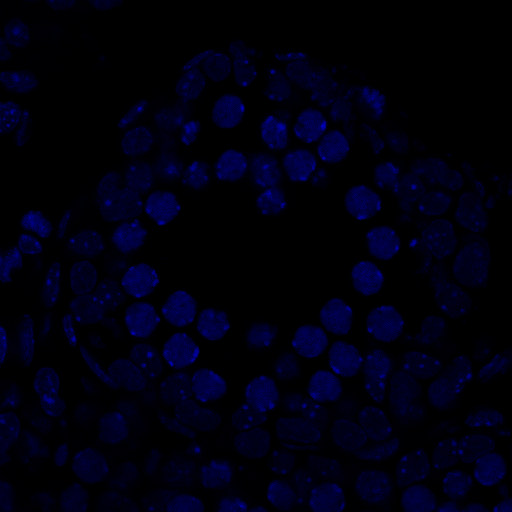

Supplement: Supplementary file 7 — Source data Fig. 1 [file 44319_2025_526_MOESM7_ESM.zip › Figure 1/1A/P15_WT/DAPI_P15_WT_Series003.png]

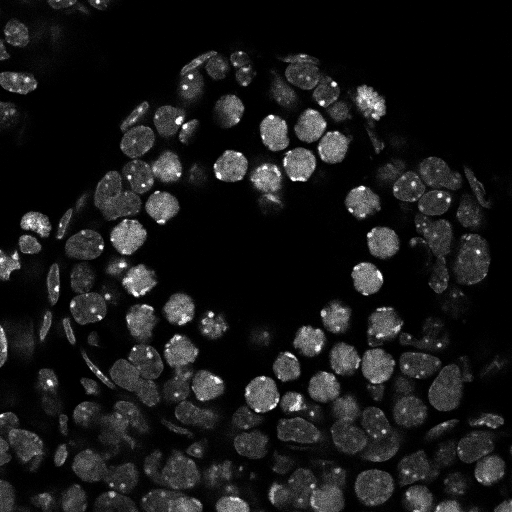

Supplement: Supplementary file 7 — Source data Fig. 1 [file 44319_2025_526_MOESM7_ESM.zip › Figure 1/1A/P15_WT/p15_D3C WT_Series003_all_channels.tif]

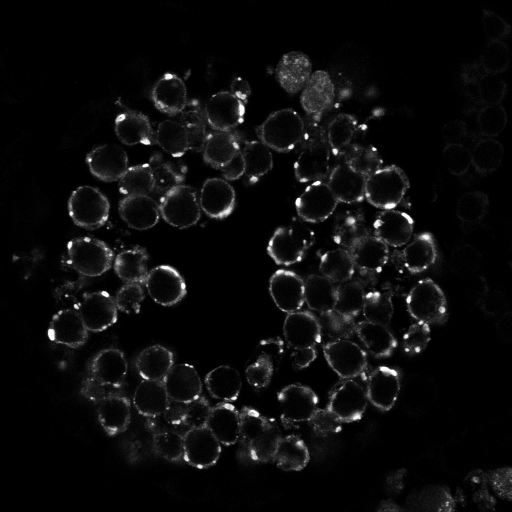

Supplement: Supplementary file 7 — Source data Fig. 1 [file 44319_2025_526_MOESM7_ESM.zip › Figure 1/1A/P20_KO/p20_D3CKO_Series001_merge.tif]

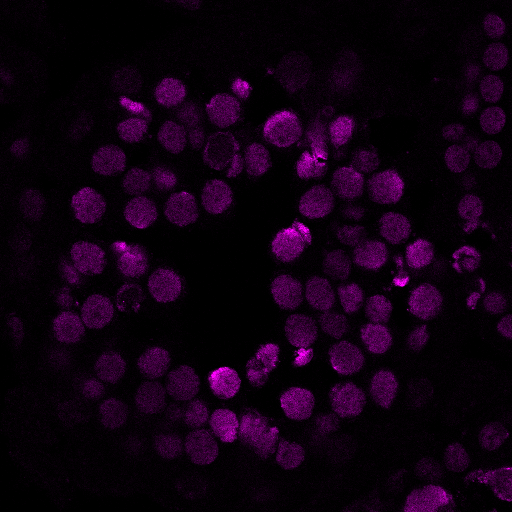

Supplement: Supplementary file 7 — Source data Fig. 1 [file 44319_2025_526_MOESM7_ESM.zip › Figure 1/1A/P20_KO/TRA98_P20_KO_Series001.png]

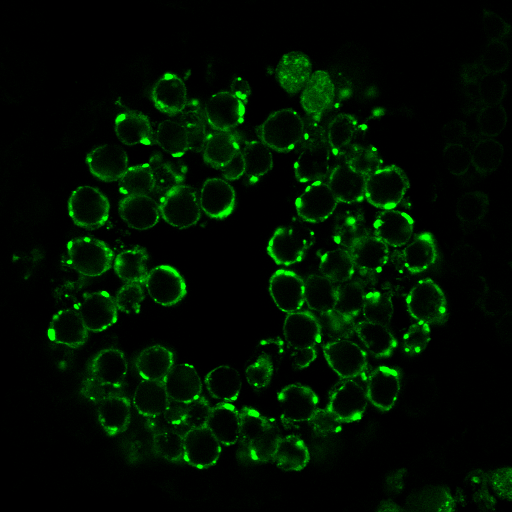

Supplement: Supplementary file 7 — Source data Fig. 1 [file 44319_2025_526_MOESM7_ESM.zip › Figure 1/1A/P20_KO/L1ORF1_P20_KO_Series001.png]

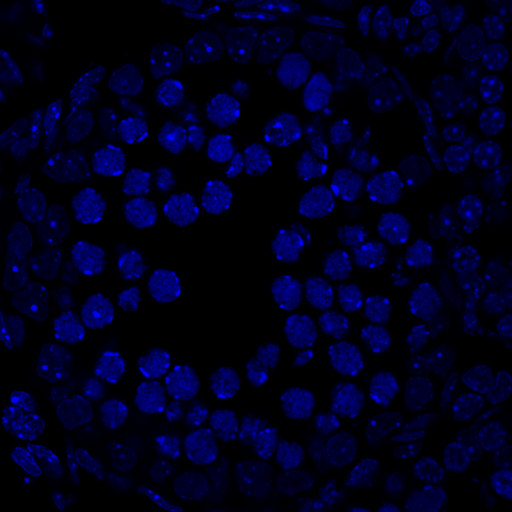

Supplement: Supplementary file 7 — Source data Fig. 1 [file 44319_2025_526_MOESM7_ESM.zip › Figure 1/1A/P20_KO/DAPI_P20_KO_Series001.png]

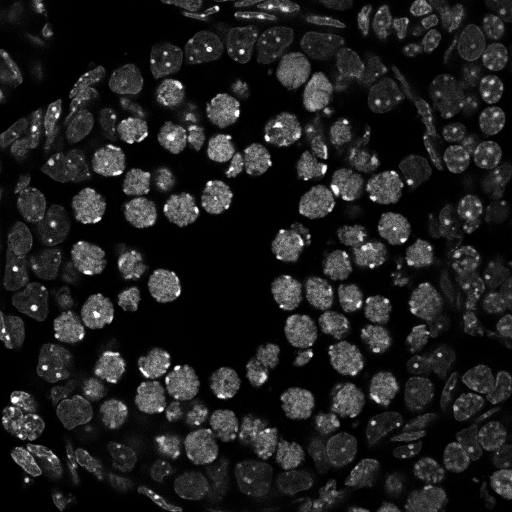

Supplement: Supplementary file 7 — Source data Fig. 1 [file 44319_2025_526_MOESM7_ESM.zip › Figure 1/1A/P20_KO/p20_D3CKO_Series001_all_channels.tif]

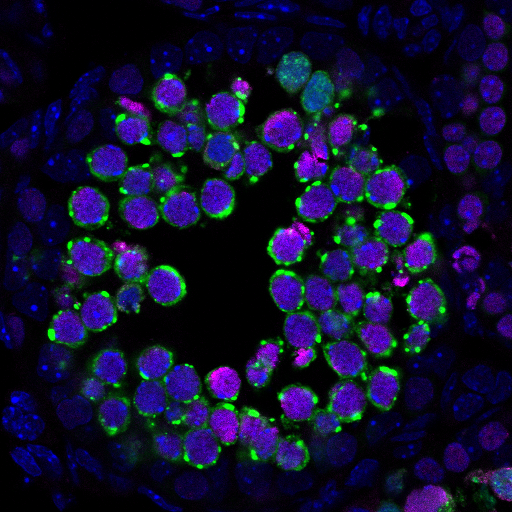

Supplement: Supplementary file 7 — Source data Fig. 1 [file 44319_2025_526_MOESM7_ESM.zip › Figure 1/1A/P20_KO/Merge-P20_KO_Series001.png]

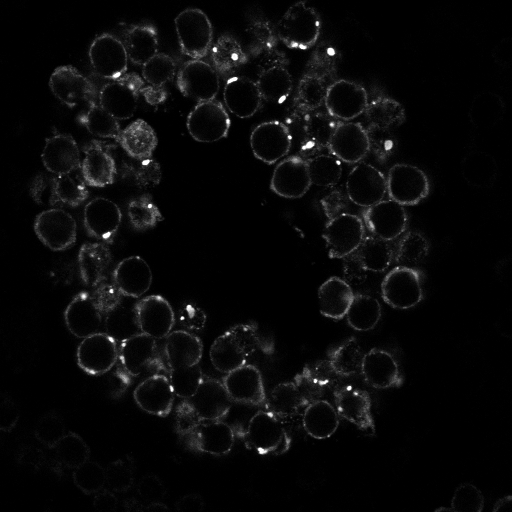

Supplement: Supplementary file 7 — Source data Fig. 1 [file 44319_2025_526_MOESM7_ESM.zip › Figure 1/1A/P30_KO/p30_D3CKO_Series001_merge.tif]

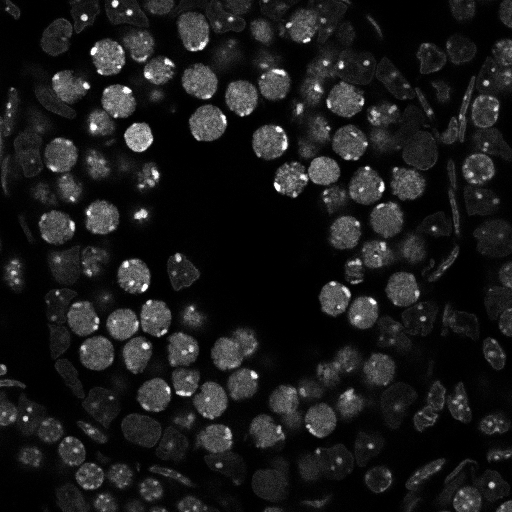

Supplement: Supplementary file 7 — Source data Fig. 1 [file 44319_2025_526_MOESM7_ESM.zip › Figure 1/1A/P30_KO/p30_D3CKO_Series001_all_channels.tif]

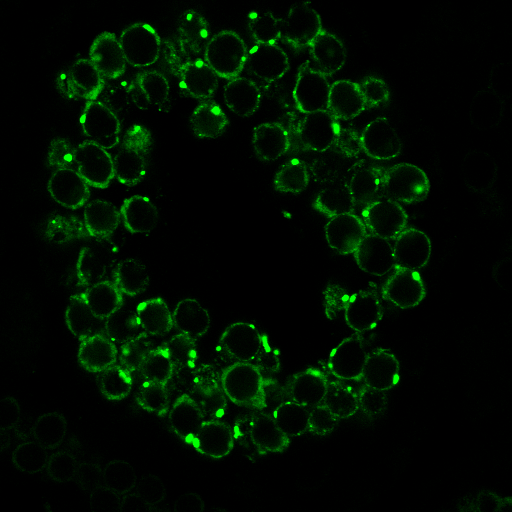

Supplement: Supplementary file 7 — Source data Fig. 1 [file 44319_2025_526_MOESM7_ESM.zip › Figure 1/1A/P30_KO/L1OR1_P30_KO_ Series001.png]

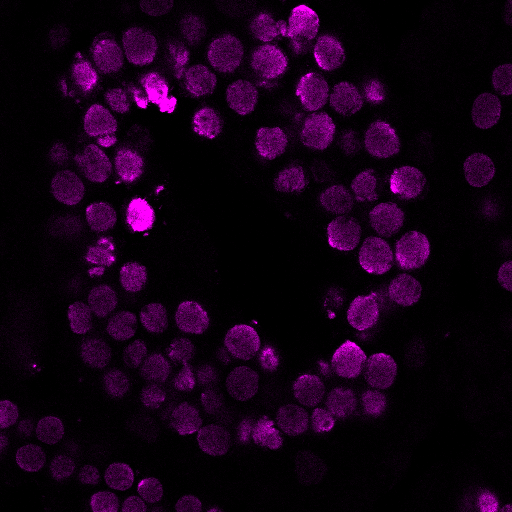

Supplement: Supplementary file 7 — Source data Fig. 1 [file 44319_2025_526_MOESM7_ESM.zip › Figure 1/1A/P30_KO/TRA98_P30_KO_Series001.png]

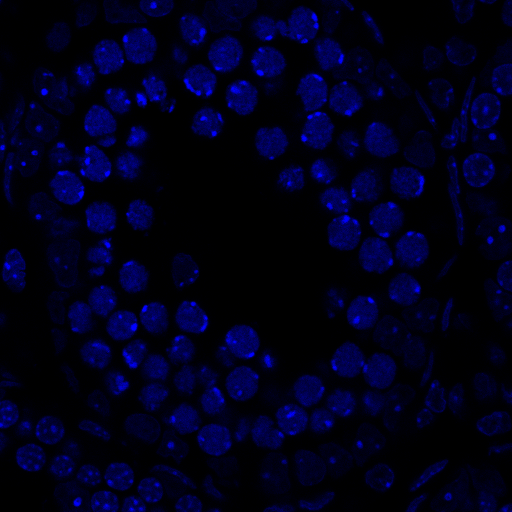

Supplement: Supplementary file 7 — Source data Fig. 1 [file 44319_2025_526_MOESM7_ESM.zip › Figure 1/1A/P30_KO/DAPI-P30_KO_Series001.png]

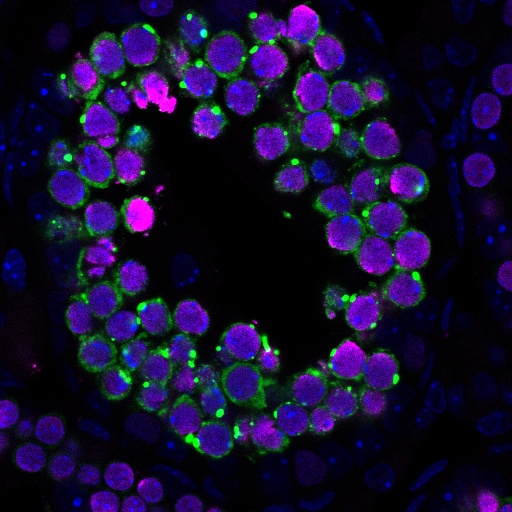

Supplement: Supplementary file 7 — Source data Fig. 1 [file 44319_2025_526_MOESM7_ESM.zip › Figure 1/1A/P30_KO/MERGE-P30_KO_Series001.png]

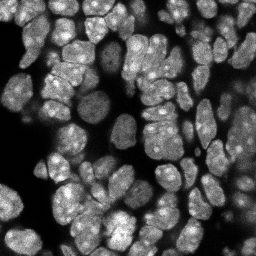

Supplement: Supplementary file 7 — Source data Fig. 1 [file 44319_2025_526_MOESM7_ESM.zip › Figure 1/1A/E18.5_KO/E18.5_KO_all_channels_Series010.tif]

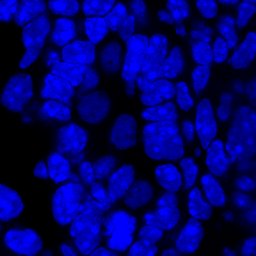

Supplement: Supplementary file 7 — Source data Fig. 1 [file 44319_2025_526_MOESM7_ESM.zip › Figure 1/1A/E18.5_KO/DAPI-E18.5_KO_ Series010.jpg]

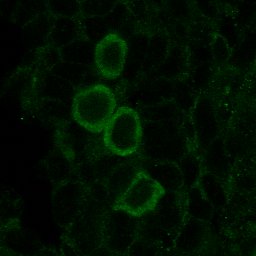

Supplement: Supplementary file 7 — Source data Fig. 1 [file 44319_2025_526_MOESM7_ESM.zip › Figure 1/1A/E18.5_KO/L1ORF1-E18.5_KO_ Series010.jpg]

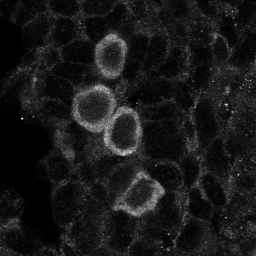

Supplement: Supplementary file 7 — Source data Fig. 1 [file 44319_2025_526_MOESM7_ESM.zip › Figure 1/1A/E18.5_KO/merge_E18.5_KO_Series010.tif]

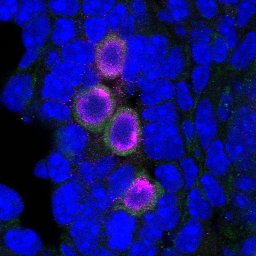

Supplement: Supplementary file 7 — Source data Fig. 1 [file 44319_2025_526_MOESM7_ESM.zip › Figure 1/1A/E18.5_KO/Merge_E18.5_KO_ Series010.jpg]

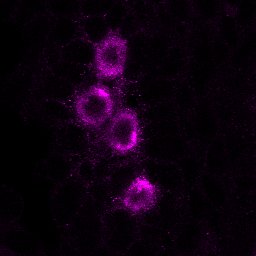

Supplement: Supplementary file 7 — Source data Fig. 1 [file 44319_2025_526_MOESM7_ESM.zip › Figure 1/1A/E18.5_KO/TRA98_E18.5_KO_ Series010.jpg]

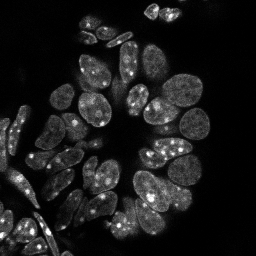

Supplement: Supplementary file 7 — Source data Fig. 1 [file 44319_2025_526_MOESM7_ESM.zip › Figure 1/1A/P5_WT/P5_WT_Series002_all_channels.tif]

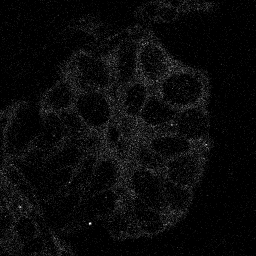

Supplement: Supplementary file 7 — Source data Fig. 1 [file 44319_2025_526_MOESM7_ESM.zip › Figure 1/1A/P5_WT/merge_P5_WT_Series002.tif]

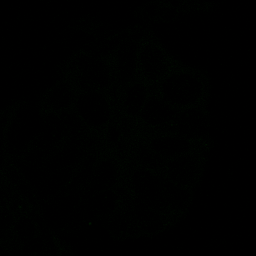

Supplement: Supplementary file 7 — Source data Fig. 1 [file 44319_2025_526_MOESM7_ESM.zip › Figure 1/1A/P5_WT/L1ORF1_P5_WT_Series002.png]

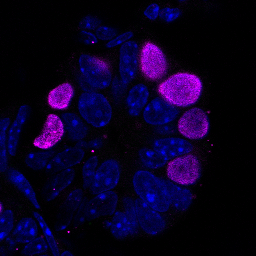

Supplement: Supplementary file 7 — Source data Fig. 1 [file 44319_2025_526_MOESM7_ESM.zip › Figure 1/1A/P5_WT/Merge_P5_WT_Series002.png]

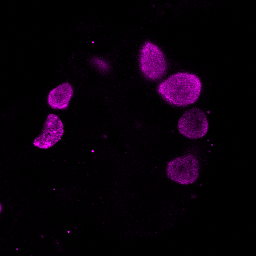

Supplement: Supplementary file 7 — Source data Fig. 1 [file 44319_2025_526_MOESM7_ESM.zip › Figure 1/1A/P5_WT/TRA98_P5_WT_Series002.png]

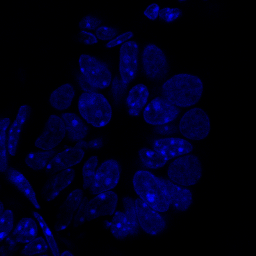

Supplement: Supplementary file 7 — Source data Fig. 1 [file 44319_2025_526_MOESM7_ESM.zip › Figure 1/1A/P5_WT/DAPI_P5_WT_Series002.png]

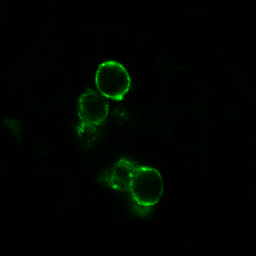

Supplement: Supplementary file 7 — Source data Fig. 1 [file 44319_2025_526_MOESM7_ESM.zip › Figure 1/1A/P10_KO/L1ORF1_P10_3CKO_Series004.png]

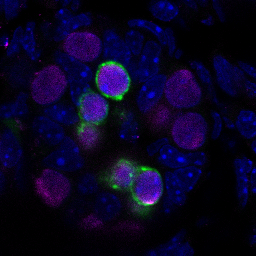

Supplement: Supplementary file 7 — Source data Fig. 1 [file 44319_2025_526_MOESM7_ESM.zip › Figure 1/1A/P10_KO/Merge_P10_3CKO_Series004.png]

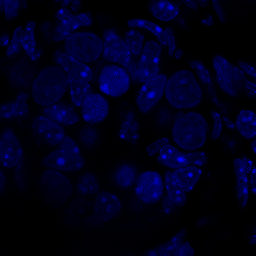

Supplement: Supplementary file 7 — Source data Fig. 1 [file 44319_2025_526_MOESM7_ESM.zip › Figure 1/1A/P10_KO/DAPI_P10_3CKO_Series004.png]

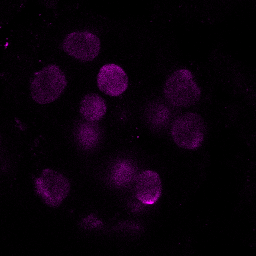

Supplement: Supplementary file 7 — Source data Fig. 1 [file 44319_2025_526_MOESM7_ESM.zip › Figure 1/1A/P10_KO/TRA98_P10_3CKO_Series004.png]

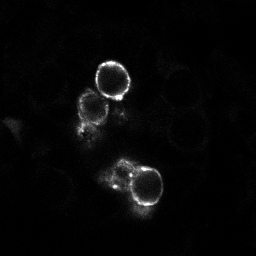

Supplement: Supplementary file 7 — Source data Fig. 1 [file 44319_2025_526_MOESM7_ESM.zip › Figure 1/1A/P10_KO/P10_3CKO_Series004_merge.tif]

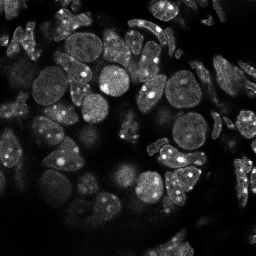

Supplement: Supplementary file 7 — Source data Fig. 1 [file 44319_2025_526_MOESM7_ESM.zip › Figure 1/1A/P10_KO/P10_3CKO_Series004_all_channels.tif]

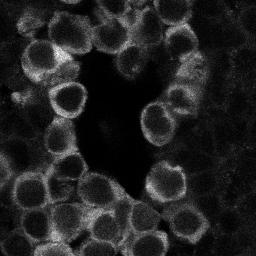

Supplement: Supplementary file 7 — Source data Fig. 1 [file 44319_2025_526_MOESM7_ESM.zip › Figure 1/1A/E15.5_KO/merge_E15.5_3CKO_Series006.tif]

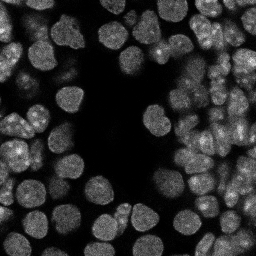

Supplement: Supplementary file 7 — Source data Fig. 1 [file 44319_2025_526_MOESM7_ESM.zip › Figure 1/1A/E15.5_KO/E15.5_3CKO_Series006_all_Channels.tif]

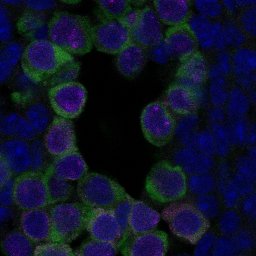

Supplement: Supplementary file 7 — Source data Fig. 1 [file 44319_2025_526_MOESM7_ESM.zip › Figure 1/1A/E15.5_KO/merge_E15.5_ Series006.jpg]

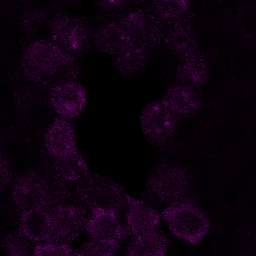

Supplement: Supplementary file 7 — Source data Fig. 1 [file 44319_2025_526_MOESM7_ESM.zip › Figure 1/1A/E15.5_KO/TRA98_E15.5_3CKO_Series006.png]

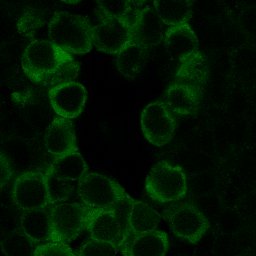

Supplement: Supplementary file 7 — Source data Fig. 1 [file 44319_2025_526_MOESM7_ESM.zip › Figure 1/1A/E15.5_KO/L1ORF1_E15.5_3CKO_ Series006.jpg]

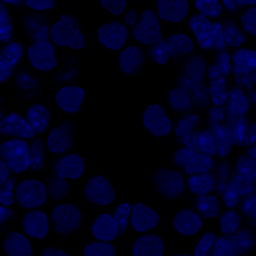

Supplement: Supplementary file 7 — Source data Fig. 1 [file 44319_2025_526_MOESM7_ESM.zip › Figure 1/1A/E15.5_KO/DAPI_E15.5_3CKO_Series006.png]

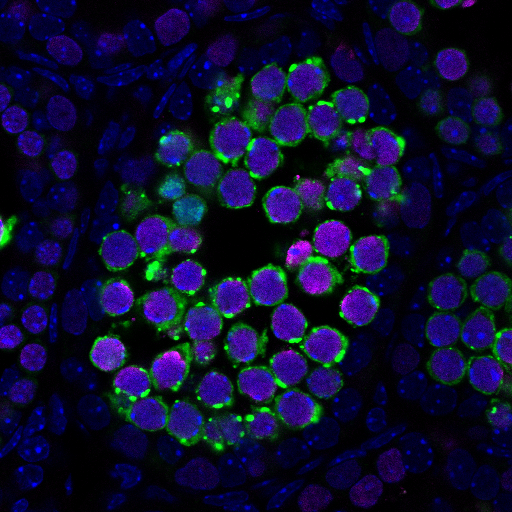

Supplement: Supplementary file 7 — Source data Fig. 1 [file 44319_2025_526_MOESM7_ESM.zip › Figure 1/1A/P15_KO/merge_P15_KO_Series012.png]

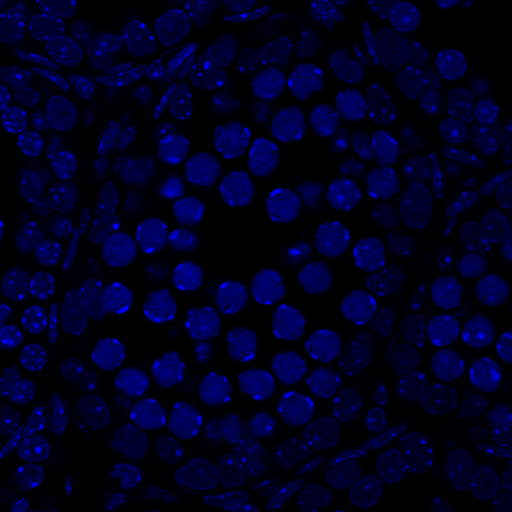

Supplement: Supplementary file 7 — Source data Fig. 1 [file 44319_2025_526_MOESM7_ESM.zip › Figure 1/1A/P15_KO/DAPI_P15_KO_Series012.png]

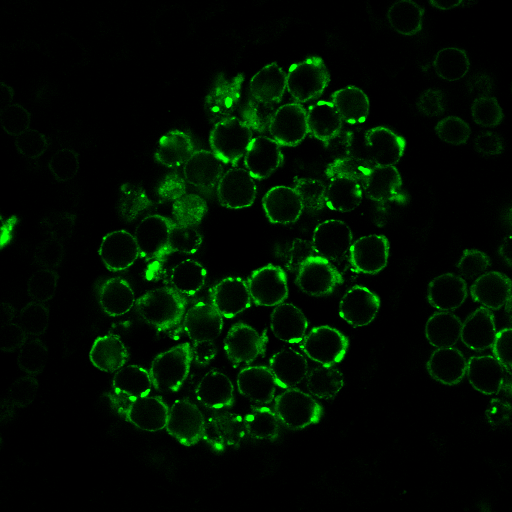

Supplement: Supplementary file 7 — Source data Fig. 1 [file 44319_2025_526_MOESM7_ESM.zip › Figure 1/1A/P15_KO/L1ORF1_P15_KO_Series012.png]

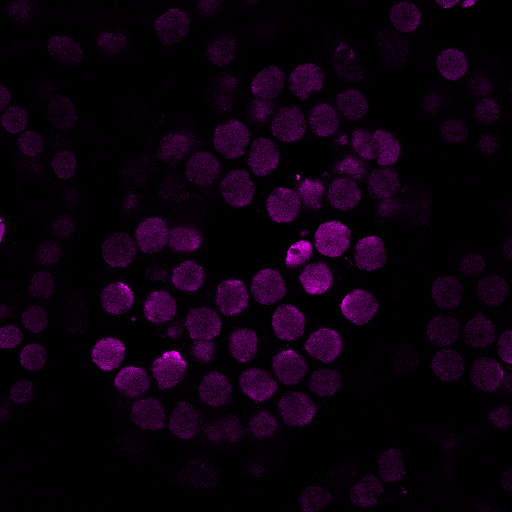

Supplement: Supplementary file 7 — Source data Fig. 1 [file 44319_2025_526_MOESM7_ESM.zip › Figure 1/1A/P15_KO/TRA98_P15_KO_Series012.png]

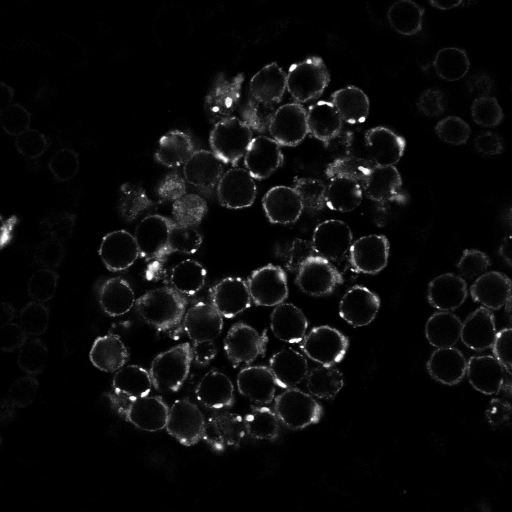

Supplement: Supplementary file 7 — Source data Fig. 1 [file 44319_2025_526_MOESM7_ESM.zip › Figure 1/1A/P15_KO/p15_D3CKO_Series012_merge.tif]

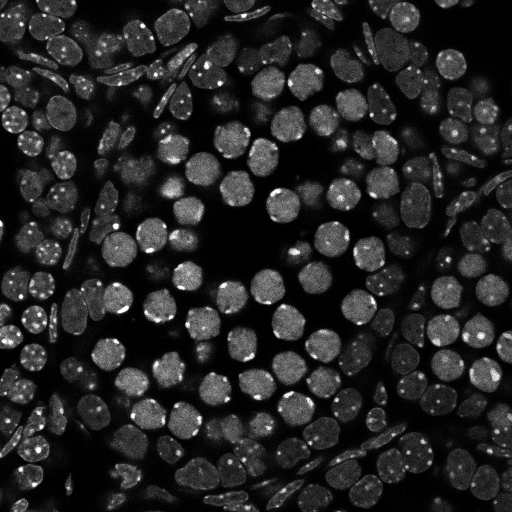

Supplement: Supplementary file 7 — Source data Fig. 1 [file 44319_2025_526_MOESM7_ESM.zip › Figure 1/1A/P15_KO/p15_D3CKO_Series012_all_channels.tif]

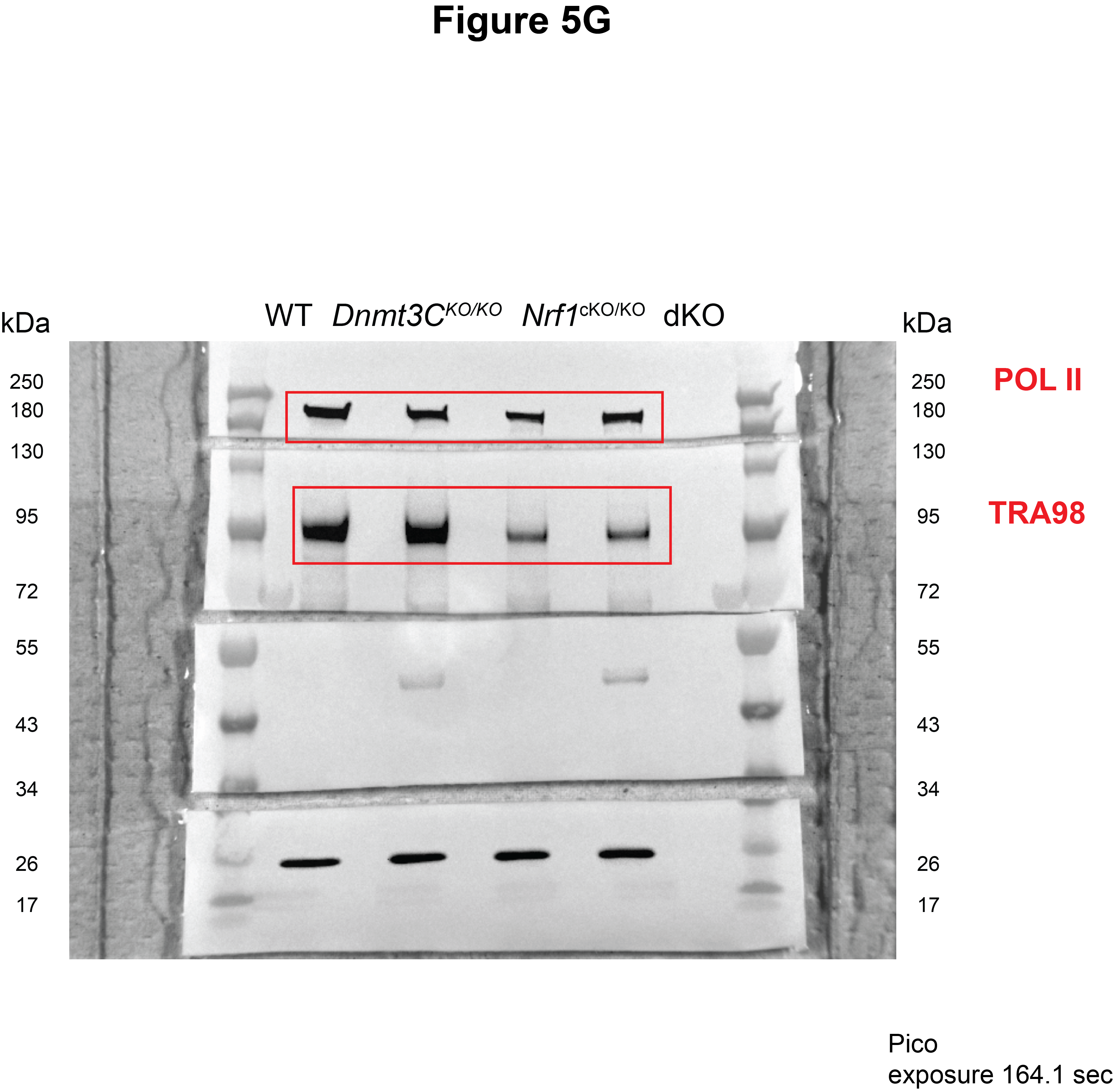

Supplement: Supplementary file 8 — Source data Fig. 5 [file 44319_2025_526_MOESM8_ESM.zip › Figure 5/5G/western_POLII-TRA98.png]

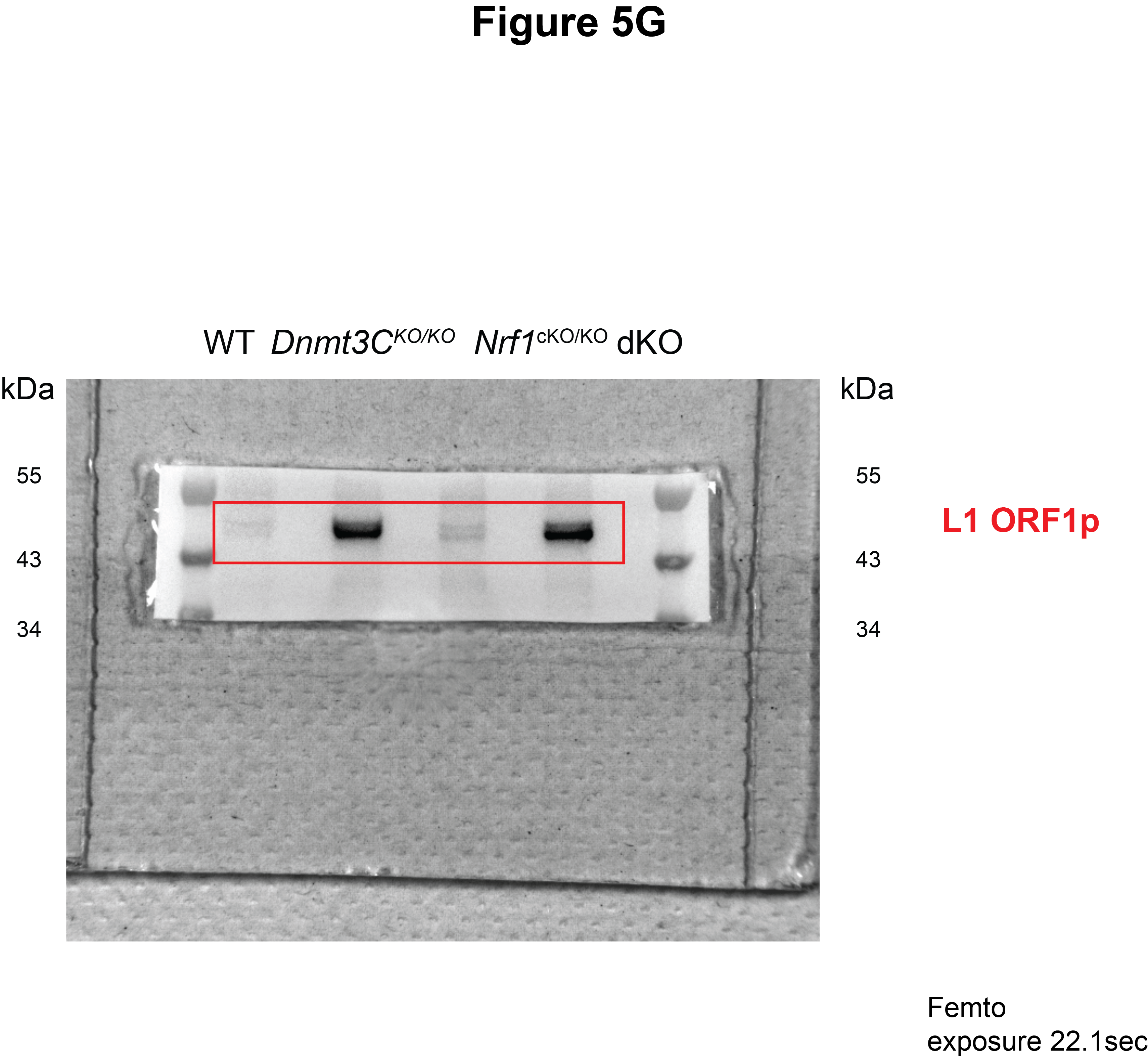

Supplement: Supplementary file 8 — Source data Fig. 5 [file 44319_2025_526_MOESM8_ESM.zip › Figure 5/5G/western_L1ORF1p.png]

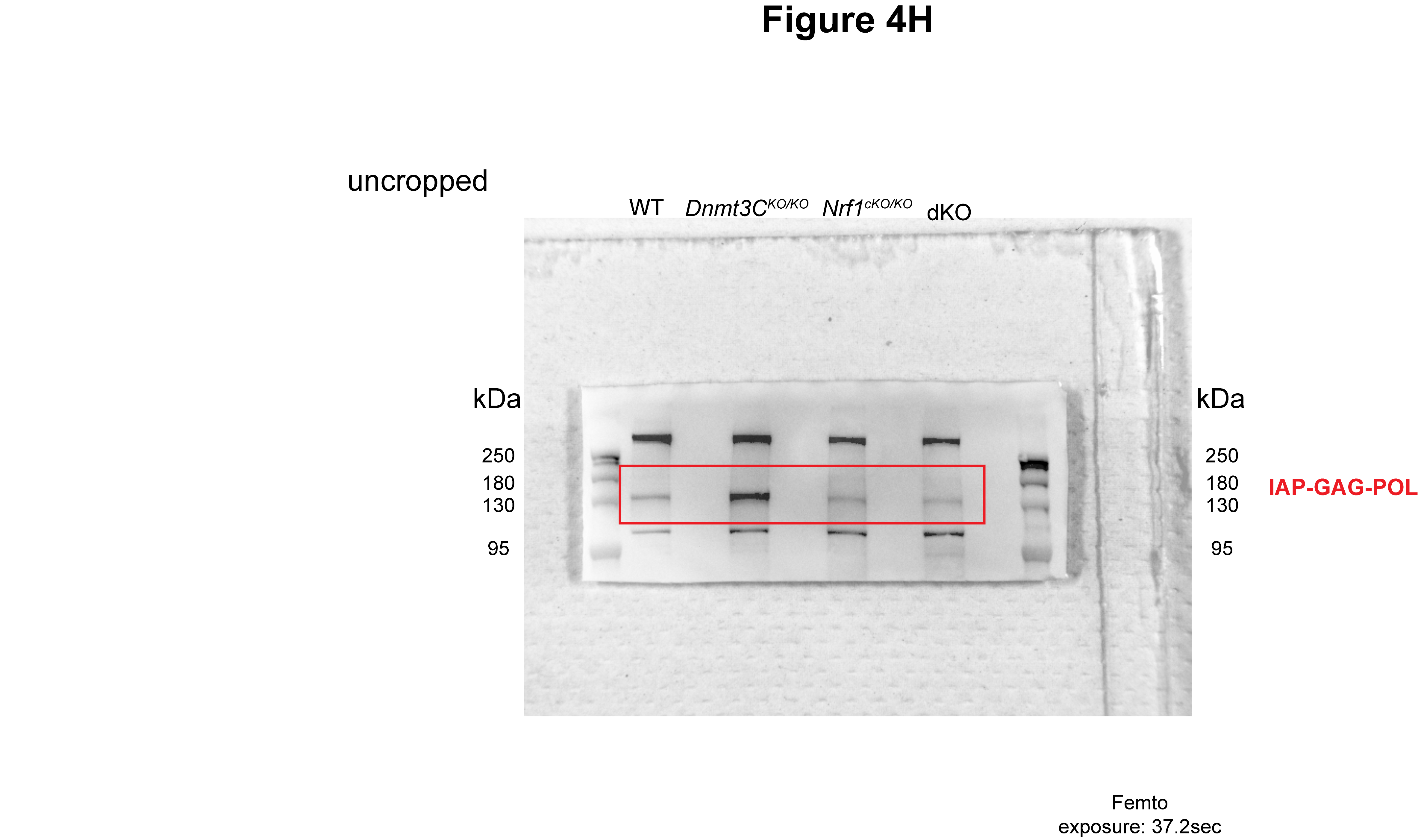

Supplement: Supplementary file 8 — Source data Fig. 5 [file 44319_2025_526_MOESM8_ESM.zip › Figure 5/5H/Western_IAP-POL-GAG.tif]

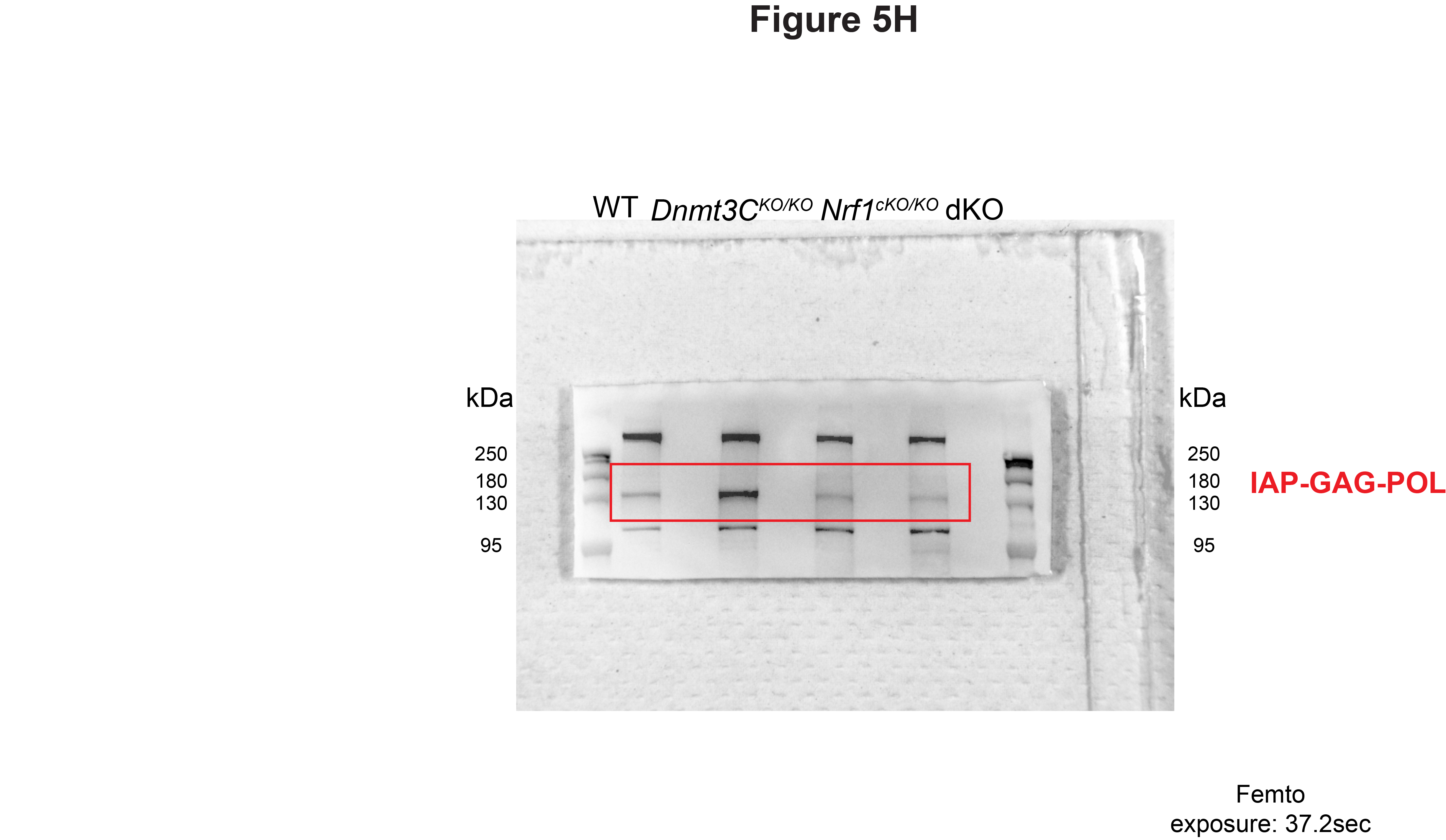

Supplement: Supplementary file 8 — Source data Fig. 5 [file 44319_2025_526_MOESM8_ESM.zip › Figure 5/5H/Western_IAP-POL-GAG.png]

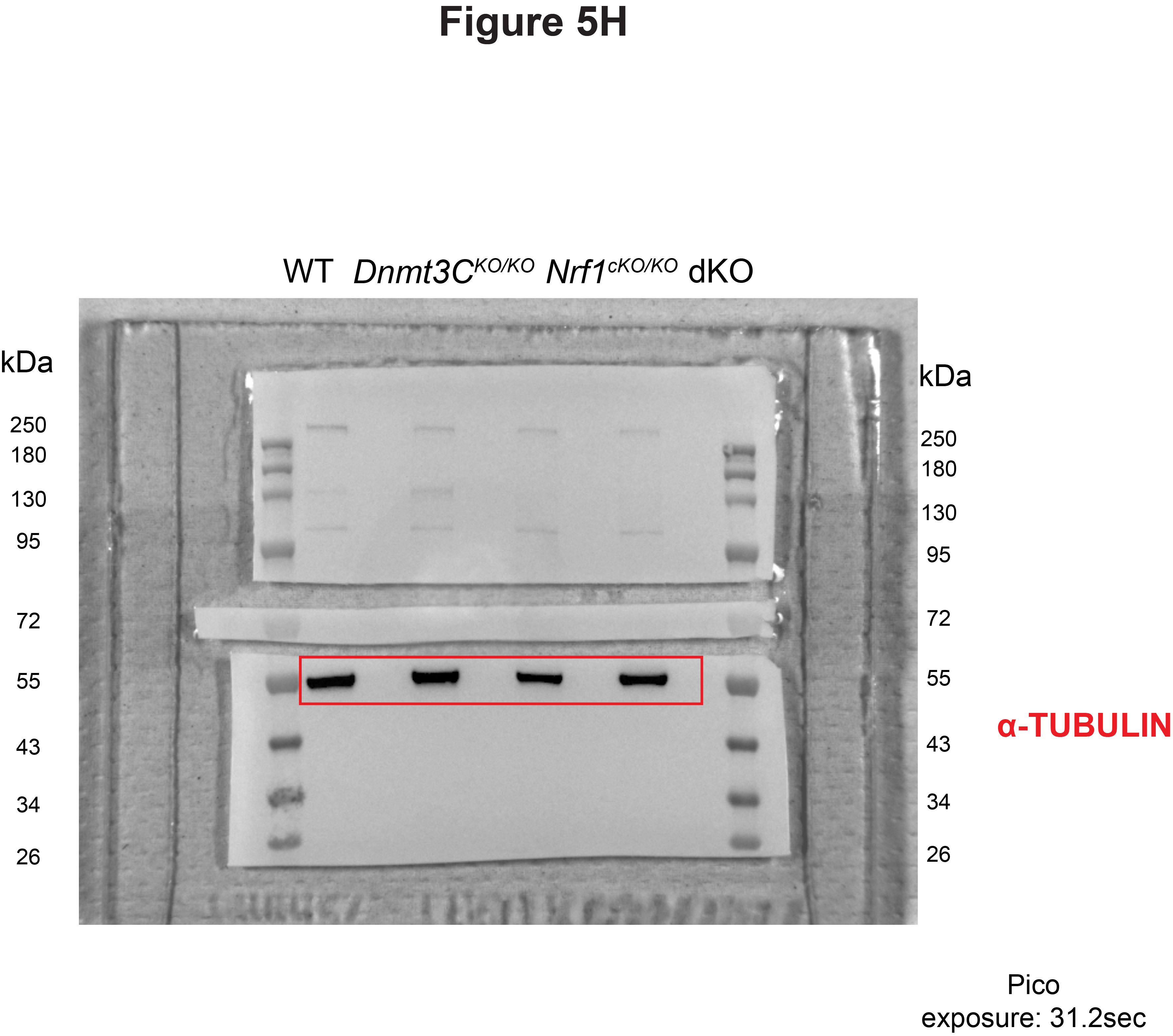

Supplement: Supplementary file 8 — Source data Fig. 5 [file 44319_2025_526_MOESM8_ESM.zip › Figure 5/5H/western_a-TUBULIN.png]

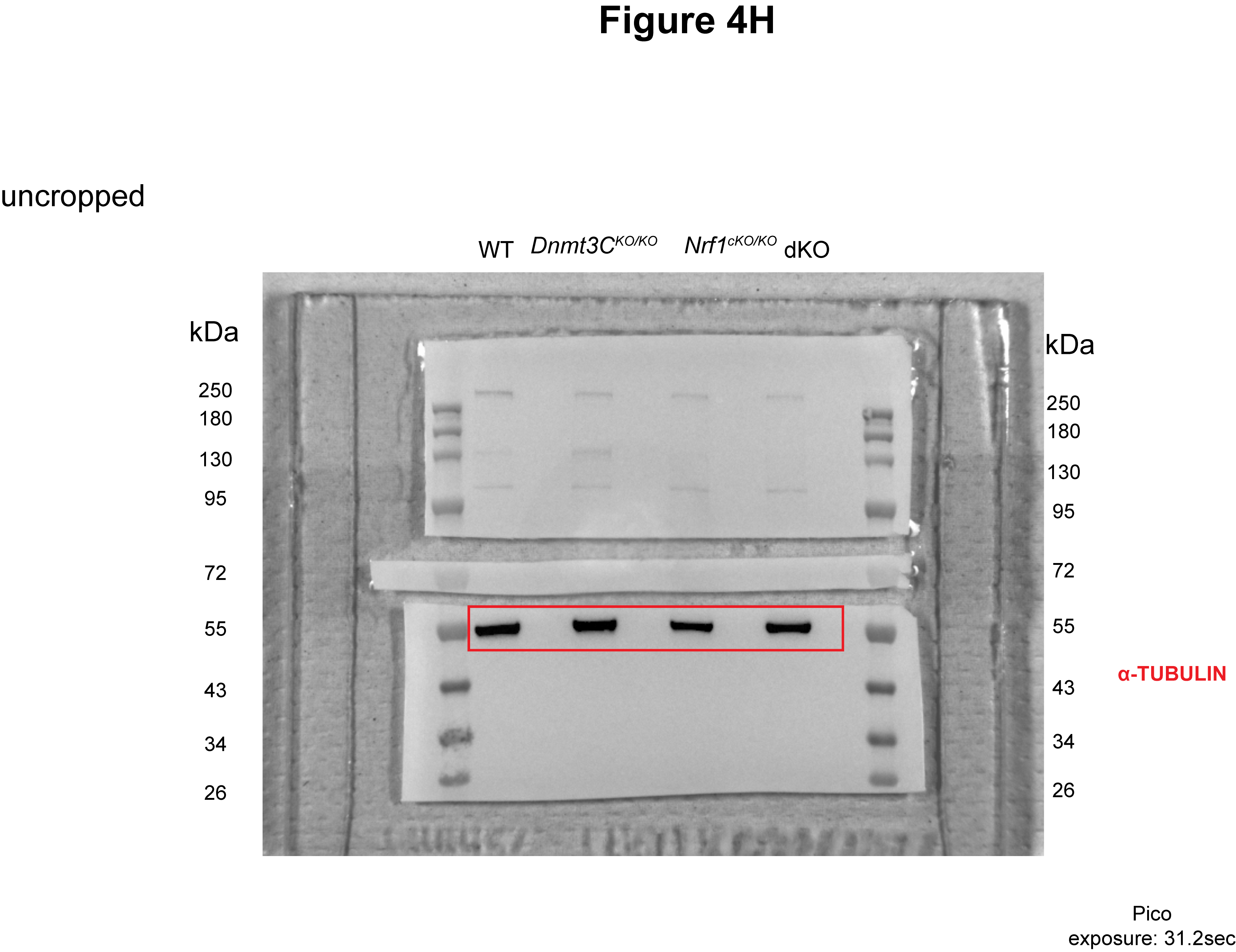

Supplement: Supplementary file 8 — Source data Fig. 5 [file 44319_2025_526_MOESM8_ESM.zip › Figure 5/5H/western_a-TUBULIN.tif]

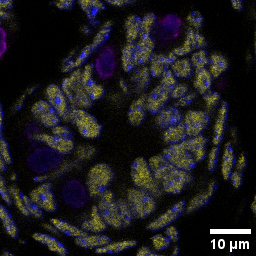

Supplement: Supplementary file 8 — Source data Fig. 5 [file 44319_2025_526_MOESM8_ESM.zip › Figure 5/5A/dKO/merge_dKO_series004.png]

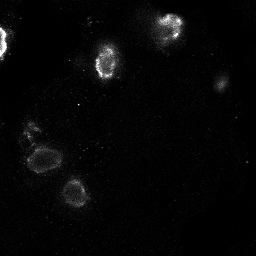

Supplement: Supplementary file 8 — Source data Fig. 5 [file 44319_2025_526_MOESM8_ESM.zip › Figure 5/5A/dKO/TRA98_dKO_Series004.tif]

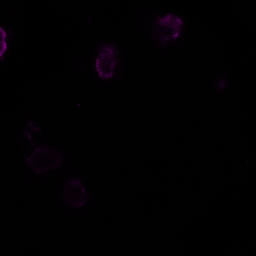

Supplement: Supplementary file 8 — Source data Fig. 5 [file 44319_2025_526_MOESM8_ESM.zip › Figure 5/5A/dKO/TRA98_dKO_Series004.png]

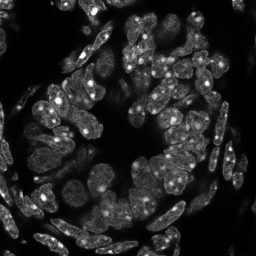

Supplement: Supplementary file 8 — Source data Fig. 5 [file 44319_2025_526_MOESM8_ESM.zip › Figure 5/5A/dKO/merge_dKO_series004.tif]

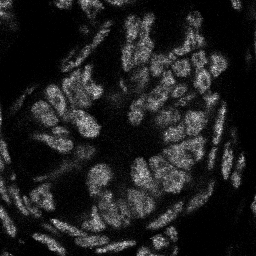

Supplement: Supplementary file 8 — Source data Fig. 5 [file 44319_2025_526_MOESM8_ESM.zip › Figure 5/5A/dKO/NRF1_dKO_Series004.tif]

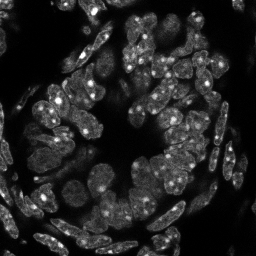

Supplement: Supplementary file 8 — Source data Fig. 5 [file 44319_2025_526_MOESM8_ESM.zip › Figure 5/5A/dKO/DAPI_dKO_ Series004.tif]

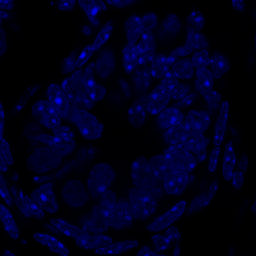

Supplement: Supplementary file 8 — Source data Fig. 5 [file 44319_2025_526_MOESM8_ESM.zip › Figure 5/5A/dKO/DAPI_dKO_ Series004.png]

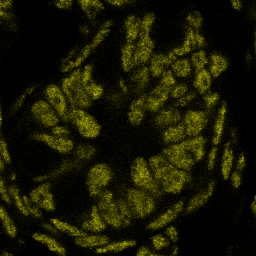

Supplement: Supplementary file 8 — Source data Fig. 5 [file 44319_2025_526_MOESM8_ESM.zip › Figure 5/5A/dKO/NRF1_dKO_Series004.png]
