# Supplementary figures and images for: DNA methylation at retrotransposons protects the germline by preventing NRF1-mediated activation (part 2 of 2)
Source: EMBO Rep. 2025 Aug 4;26(17):4312–39. doi: 10.1038/s44319-025-00526-1 (PMC12420836; doi:10.1038/s44319-025-00526-1)

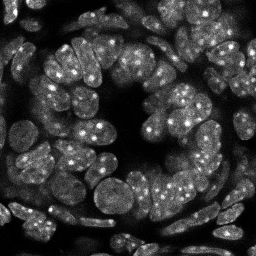

Supplement: Supplementary file 8 — Source data Fig. 5 [file 44319_2025_526_MOESM8_ESM.zip › Figure 5/5A/Nrf1cKO/merge_Nrf1cKO_Series004.tif]

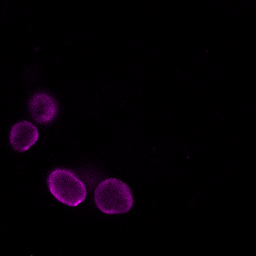

Supplement: Supplementary file 8 — Source data Fig. 5 [file 44319_2025_526_MOESM8_ESM.zip › Figure 5/5A/Nrf1cKO/TRA98_Nrf1cKO_Series004.png]

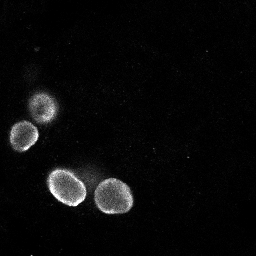

Supplement: Supplementary file 8 — Source data Fig. 5 [file 44319_2025_526_MOESM8_ESM.zip › Figure 5/5A/Nrf1cKO/TRA98_Nrf1cKO_Series004.tif]

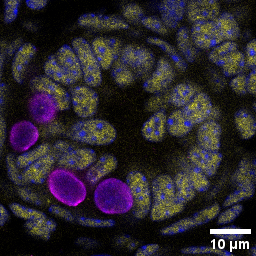

Supplement: Supplementary file 8 — Source data Fig. 5 [file 44319_2025_526_MOESM8_ESM.zip › Figure 5/5A/Nrf1cKO/merge_Nrf1cKO_Series004.png]

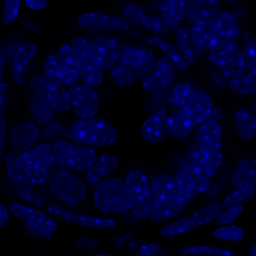

Supplement: Supplementary file 8 — Source data Fig. 5 [file 44319_2025_526_MOESM8_ESM.zip › Figure 5/5A/Nrf1cKO/DAPI_Nrf1cKO_Series004.png]

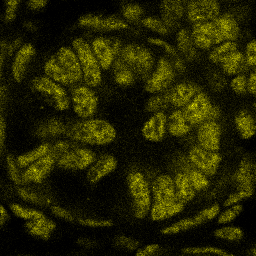

Supplement: Supplementary file 8 — Source data Fig. 5 [file 44319_2025_526_MOESM8_ESM.zip › Figure 5/5A/Nrf1cKO/NRF1_Nrf1cKO_Series004.png]

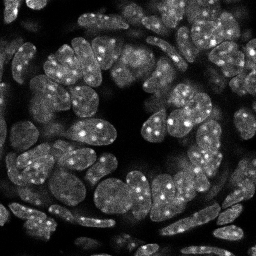

Supplement: Supplementary file 8 — Source data Fig. 5 [file 44319_2025_526_MOESM8_ESM.zip › Figure 5/5A/Nrf1cKO/DAPI_Nrf1cKO_Series004.tif]

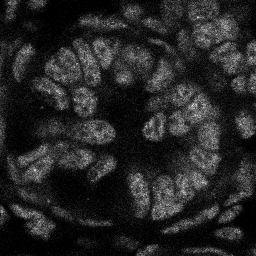

Supplement: Supplementary file 8 — Source data Fig. 5 [file 44319_2025_526_MOESM8_ESM.zip › Figure 5/5A/Nrf1cKO/NRF1_Nrf1cKO_Series004.tif]

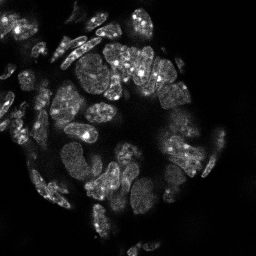

Supplement: Supplementary file 8 — Source data Fig. 5 [file 44319_2025_526_MOESM8_ESM.zip › Figure 5/5A/WT/DAPI_WT_Series002.tif]

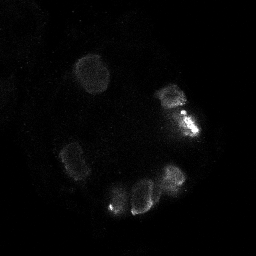

Supplement: Supplementary file 8 — Source data Fig. 5 [file 44319_2025_526_MOESM8_ESM.zip › Figure 5/5A/WT/TRA98_WT_ Series002.tif]

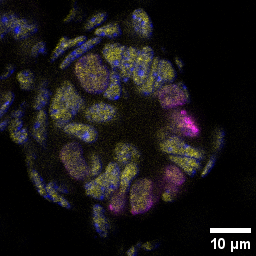

Supplement: Supplementary file 8 — Source data Fig. 5 [file 44319_2025_526_MOESM8_ESM.zip › Figure 5/5A/WT/Merge_WT_ Series002.png]

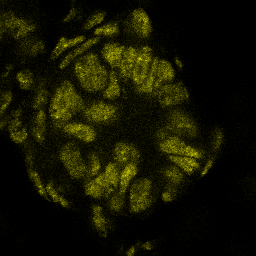

Supplement: Supplementary file 8 — Source data Fig. 5 [file 44319_2025_526_MOESM8_ESM.zip › Figure 5/5A/WT/NRF1_WT_Series002.png]

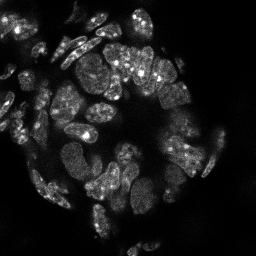

Supplement: Supplementary file 8 — Source data Fig. 5 [file 44319_2025_526_MOESM8_ESM.zip › Figure 5/5A/WT/Merge_WT_ Series002.tif]

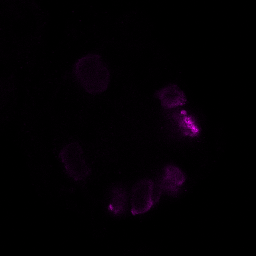

Supplement: Supplementary file 8 — Source data Fig. 5 [file 44319_2025_526_MOESM8_ESM.zip › Figure 5/5A/WT/TRA98_WT_ Series002.png]

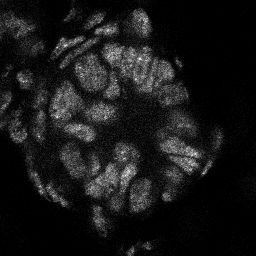

Supplement: Supplementary file 8 — Source data Fig. 5 [file 44319_2025_526_MOESM8_ESM.zip › Figure 5/5A/WT/NRF1_WT_Series002.tif]

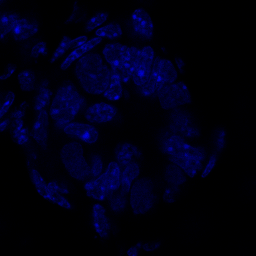

Supplement: Supplementary file 8 — Source data Fig. 5 [file 44319_2025_526_MOESM8_ESM.zip › Figure 5/5A/WT/DAPI_WT_Series002.png]

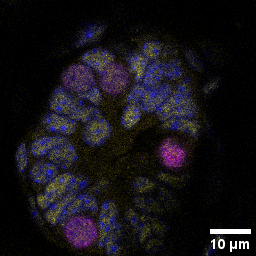

Supplement: Supplementary file 8 — Source data Fig. 5 [file 44319_2025_526_MOESM8_ESM.zip › Figure 5/5A/Dnmt3C_KO/merge_Dnmt3CKO_Series11.png]

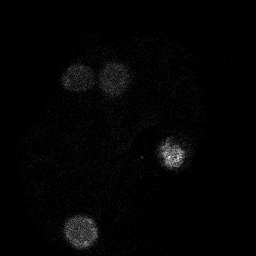

Supplement: Supplementary file 8 — Source data Fig. 5 [file 44319_2025_526_MOESM8_ESM.zip › Figure 5/5A/Dnmt3C_KO/TRA98_Dnmt3CKO_Series11.tif]

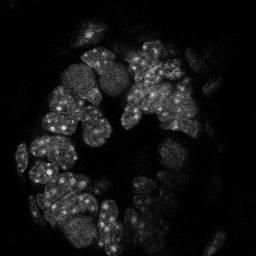

Supplement: Supplementary file 8 — Source data Fig. 5 [file 44319_2025_526_MOESM8_ESM.zip › Figure 5/5A/Dnmt3C_KO/DAPI_Dnmt3C_Series11.tif]

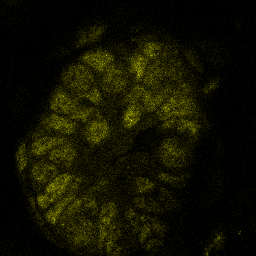

Supplement: Supplementary file 8 — Source data Fig. 5 [file 44319_2025_526_MOESM8_ESM.zip › Figure 5/5A/Dnmt3C_KO/NRF1_Dntm3CKO_Series11.png]

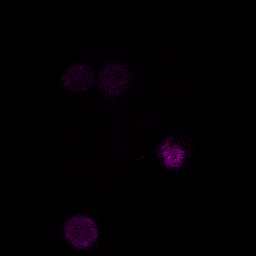

Supplement: Supplementary file 8 — Source data Fig. 5 [file 44319_2025_526_MOESM8_ESM.zip › Figure 5/5A/Dnmt3C_KO/TRA98_Dnmt3CKO_Series11.png]

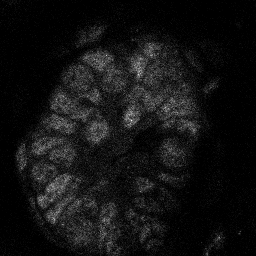

Supplement: Supplementary file 8 — Source data Fig. 5 [file 44319_2025_526_MOESM8_ESM.zip › Figure 5/5A/Dnmt3C_KO/NRF1_Dntm3CKO_Series11.tif]

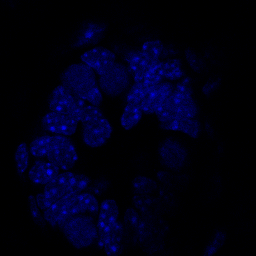

Supplement: Supplementary file 8 — Source data Fig. 5 [file 44319_2025_526_MOESM8_ESM.zip › Figure 5/5A/Dnmt3C_KO/DAPI_Dnmt3C_Series11.png]

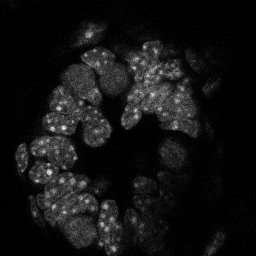

Supplement: Supplementary file 8 — Source data Fig. 5 [file 44319_2025_526_MOESM8_ESM.zip › Figure 5/5A/Dnmt3C_KO/merge_Dnmt3CKO_Series11.tif]

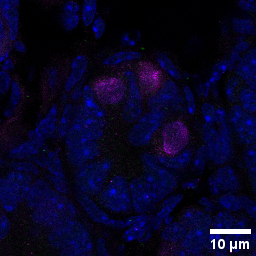

Supplement: Supplementary file 8 — Source data Fig. 5 [file 44319_2025_526_MOESM8_ESM.zip › Figure 5/5F/dKO/merge_dKO_Series002.png]

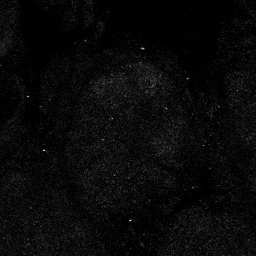

Supplement: Supplementary file 8 — Source data Fig. 5 [file 44319_2025_526_MOESM8_ESM.zip › Figure 5/5F/dKO/IAP_dKO_Series002.tif]

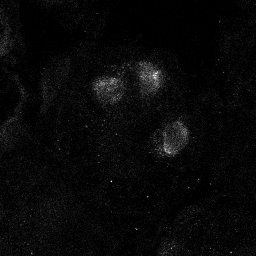

Supplement: Supplementary file 8 — Source data Fig. 5 [file 44319_2025_526_MOESM8_ESM.zip › Figure 5/5F/dKO/TRA98_dKO_Series002.tif]

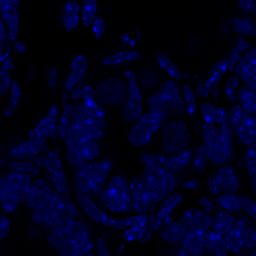

Supplement: Supplementary file 8 — Source data Fig. 5 [file 44319_2025_526_MOESM8_ESM.zip › Figure 5/5F/dKO/DAPI_dKO_Series002.png]

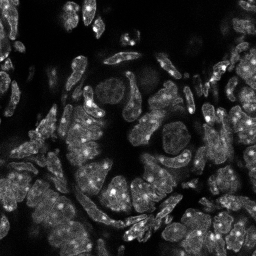

Supplement: Supplementary file 8 — Source data Fig. 5 [file 44319_2025_526_MOESM8_ESM.zip › Figure 5/5F/dKO/DAPI_dKO_Series002.tif]

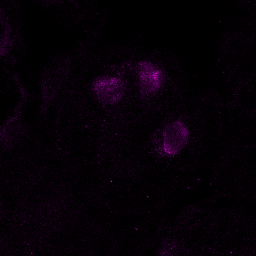

Supplement: Supplementary file 8 — Source data Fig. 5 [file 44319_2025_526_MOESM8_ESM.zip › Figure 5/5F/dKO/TRA98_dKO_Series002.png]

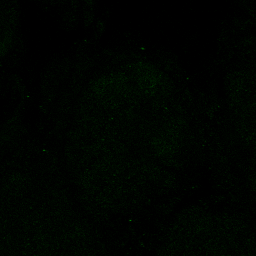

Supplement: Supplementary file 8 — Source data Fig. 5 [file 44319_2025_526_MOESM8_ESM.zip › Figure 5/5F/dKO/IAP_dKO_Series002.png]

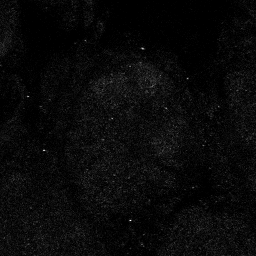

Supplement: Supplementary file 8 — Source data Fig. 5 [file 44319_2025_526_MOESM8_ESM.zip › Figure 5/5F/dKO/merge_dKO_Series002.tif]

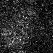

Supplement: Supplementary file 8 — Source data Fig. 5 [file 44319_2025_526_MOESM8_ESM.zip › Figure 5/5F/dKO/merge_crop_dKO_Series002.tif]

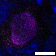

Supplement: Supplementary file 8 — Source data Fig. 5 [file 44319_2025_526_MOESM8_ESM.zip › Figure 5/5F/dKO/merge_crop_dKO_Series002.png]

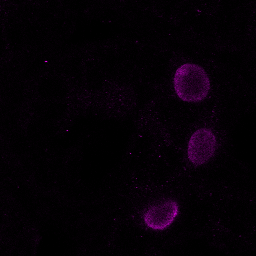

Supplement: Supplementary file 8 — Source data Fig. 5 [file 44319_2025_526_MOESM8_ESM.zip › Figure 5/5F/Nrf1cKO/TRA98_Nrf1cKO_Series002.png]

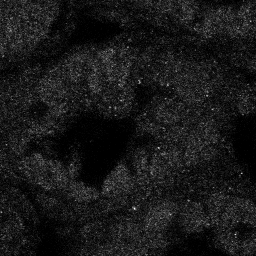

Supplement: Supplementary file 8 — Source data Fig. 5 [file 44319_2025_526_MOESM8_ESM.zip › Figure 5/5F/Nrf1cKO/merge_Nrf1cKO_Series002.tif]

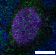

Supplement: Supplementary file 8 — Source data Fig. 5 [file 44319_2025_526_MOESM8_ESM.zip › Figure 5/5F/Nrf1cKO/merge_crop_Nrf1cKO_Series002.png]

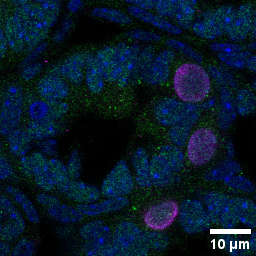

Supplement: Supplementary file 8 — Source data Fig. 5 [file 44319_2025_526_MOESM8_ESM.zip › Figure 5/5F/Nrf1cKO/merge_Nrf1cKO_Series002.png]

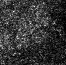

Supplement: Supplementary file 8 — Source data Fig. 5 [file 44319_2025_526_MOESM8_ESM.zip › Figure 5/5F/Nrf1cKO/merge_crop_Nrf1cKO_Series002.tif]

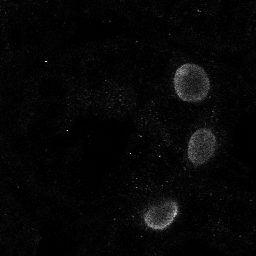

Supplement: Supplementary file 8 — Source data Fig. 5 [file 44319_2025_526_MOESM8_ESM.zip › Figure 5/5F/Nrf1cKO/TRA98_Nrf1cKO_Series002.tif]

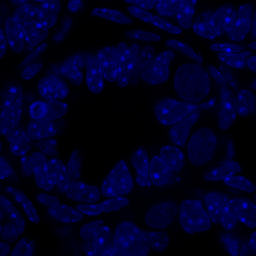

Supplement: Supplementary file 8 — Source data Fig. 5 [file 44319_2025_526_MOESM8_ESM.zip › Figure 5/5F/Nrf1cKO/DAPI_Nrf1cKO_Series002.png]

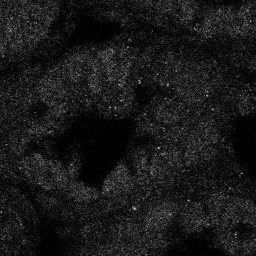

Supplement: Supplementary file 8 — Source data Fig. 5 [file 44319_2025_526_MOESM8_ESM.zip › Figure 5/5F/Nrf1cKO/IAP_Nrf1cKO_Series002_zoom3-1.tif]

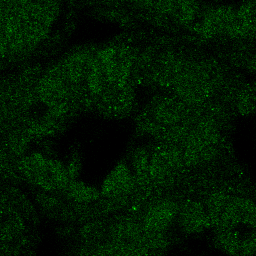

Supplement: Supplementary file 8 — Source data Fig. 5 [file 44319_2025_526_MOESM8_ESM.zip › Figure 5/5F/Nrf1cKO/IAP_Nrf1cKO_Series002.png]

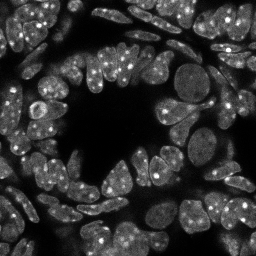

Supplement: Supplementary file 8 — Source data Fig. 5 [file 44319_2025_526_MOESM8_ESM.zip › Figure 5/5F/Nrf1cKO/DAPI_Nrf1cKO_Series002.tif]

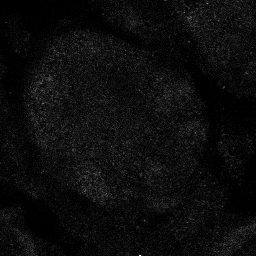

Supplement: Supplementary file 8 — Source data Fig. 5 [file 44319_2025_526_MOESM8_ESM.zip › Figure 5/5F/WT/IAP-POL_WT_ Series001.tif]

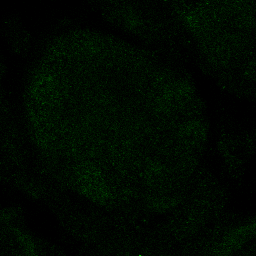

Supplement: Supplementary file 8 — Source data Fig. 5 [file 44319_2025_526_MOESM8_ESM.zip › Figure 5/5F/WT/IAP-POL_WT_ Series001.png]

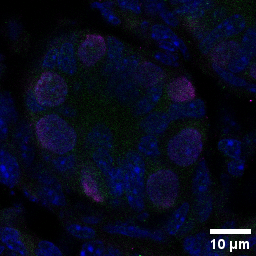

Supplement: Supplementary file 8 — Source data Fig. 5 [file 44319_2025_526_MOESM8_ESM.zip › Figure 5/5F/WT/merge_WT_Series001.png]

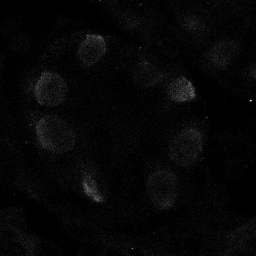

Supplement: Supplementary file 8 — Source data Fig. 5 [file 44319_2025_526_MOESM8_ESM.zip › Figure 5/5F/WT/TRA98_WT_Series001.tif]

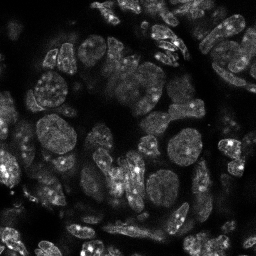

Supplement: Supplementary file 8 — Source data Fig. 5 [file 44319_2025_526_MOESM8_ESM.zip › Figure 5/5F/WT/DAPI_WT_Series001.tif]

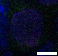

Supplement: Supplementary file 8 — Source data Fig. 5 [file 44319_2025_526_MOESM8_ESM.zip › Figure 5/5F/WT/merge_crop_WT_Series001.png]

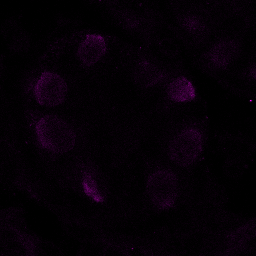

Supplement: Supplementary file 8 — Source data Fig. 5 [file 44319_2025_526_MOESM8_ESM.zip › Figure 5/5F/WT/TRA98_WT_Series001.png]

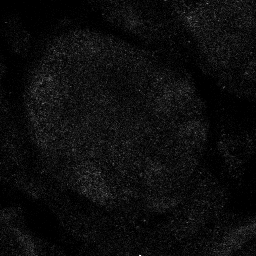

Supplement: Supplementary file 8 — Source data Fig. 5 [file 44319_2025_526_MOESM8_ESM.zip › Figure 5/5F/WT/merge_WT_Series001.tif]

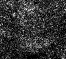

Supplement: Supplementary file 8 — Source data Fig. 5 [file 44319_2025_526_MOESM8_ESM.zip › Figure 5/5F/WT/merge_crop_WT_Series001.tif]

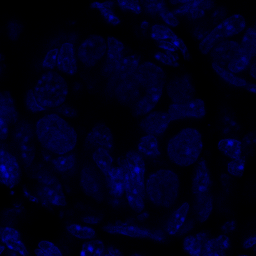

Supplement: Supplementary file 8 — Source data Fig. 5 [file 44319_2025_526_MOESM8_ESM.zip › Figure 5/5F/WT/DAPI_WT_Series001.png]

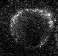

Supplement: Supplementary file 8 — Source data Fig. 5 [file 44319_2025_526_MOESM8_ESM.zip › Figure 5/5F/Dnmt3C_KO/merge_crop_Dnmt3CKO_Series003.tif]

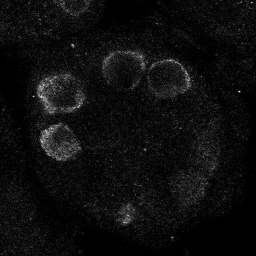

Supplement: Supplementary file 8 — Source data Fig. 5 [file 44319_2025_526_MOESM8_ESM.zip › Figure 5/5F/Dnmt3C_KO/merge_Dnmt3CKO_Series003.tif]

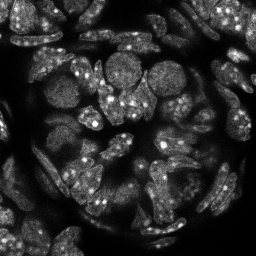

Supplement: Supplementary file 8 — Source data Fig. 5 [file 44319_2025_526_MOESM8_ESM.zip › Figure 5/5F/Dnmt3C_KO/DAPI_Dnmt3CKO_Series003.tif]

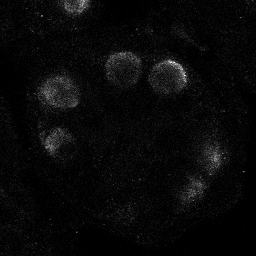

Supplement: Supplementary file 8 — Source data Fig. 5 [file 44319_2025_526_MOESM8_ESM.zip › Figure 5/5F/Dnmt3C_KO/TRA98_Dnmt3CKO_Series003.tif]

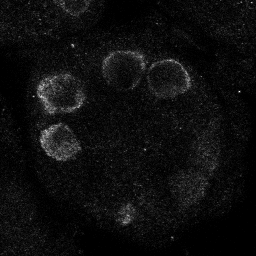

Supplement: Supplementary file 8 — Source data Fig. 5 [file 44319_2025_526_MOESM8_ESM.zip › Figure 5/5F/Dnmt3C_KO/IAP_Dnmt3CKO_Series003.tif]

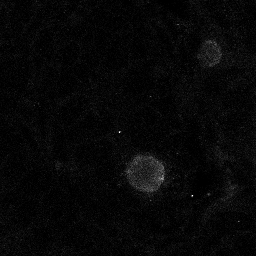

Supplement: Supplementary file 8 — Source data Fig. 5 [file 44319_2025_526_MOESM8_ESM.zip › Figure 5/5E/dKO/TRA98_dKO_Series003.tif]

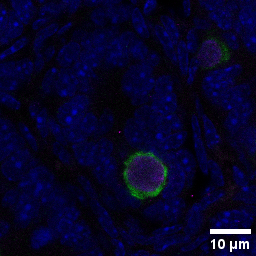

Supplement: Supplementary file 8 — Source data Fig. 5 [file 44319_2025_526_MOESM8_ESM.zip › Figure 5/5E/dKO/merge_dKO_Series003.png]

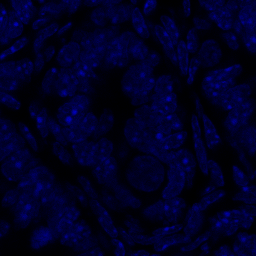

Supplement: Supplementary file 8 — Source data Fig. 5 [file 44319_2025_526_MOESM8_ESM.zip › Figure 5/5E/dKO/DAPI_dKO_Series003.png]

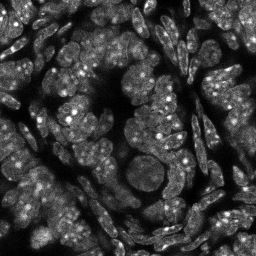

Supplement: Supplementary file 8 — Source data Fig. 5 [file 44319_2025_526_MOESM8_ESM.zip › Figure 5/5E/dKO/DAPI_dKO_Series003.tif]

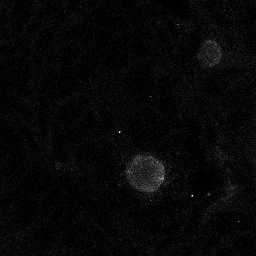

Supplement: Supplementary file 8 — Source data Fig. 5 [file 44319_2025_526_MOESM8_ESM.zip › Figure 5/5E/dKO/merge_dKO_Series003.tif]

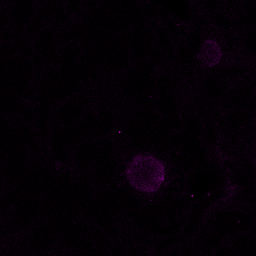

Supplement: Supplementary file 8 — Source data Fig. 5 [file 44319_2025_526_MOESM8_ESM.zip › Figure 5/5E/dKO/TRA98_dKO_Series003.png]

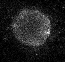

Supplement: Supplementary file 8 — Source data Fig. 5 [file 44319_2025_526_MOESM8_ESM.zip › Figure 5/5E/dKO/merge_crop_dKO_Series003.tif]

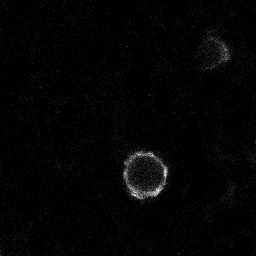

Supplement: Supplementary file 8 — Source data Fig. 5 [file 44319_2025_526_MOESM8_ESM.zip › Figure 5/5E/dKO/L1ORF1p_dKO_zoom3.tif]

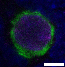

Supplement: Supplementary file 8 — Source data Fig. 5 [file 44319_2025_526_MOESM8_ESM.zip › Figure 5/5E/dKO/merge_crop_dKO_Series003.png]

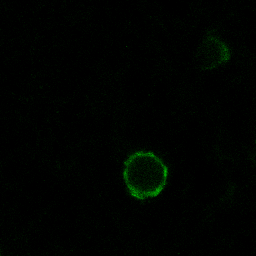

Supplement: Supplementary file 8 — Source data Fig. 5 [file 44319_2025_526_MOESM8_ESM.zip › Figure 5/5E/dKO/L1ORF1p_dKO_zoom3.png]

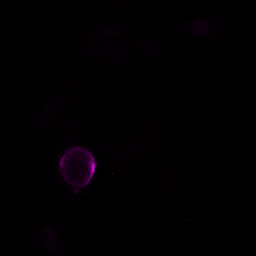

Supplement: Supplementary file 8 — Source data Fig. 5 [file 44319_2025_526_MOESM8_ESM.zip › Figure 5/5E/Nrf1cKO/TRA98_Nrf1cKO_Series002.png]

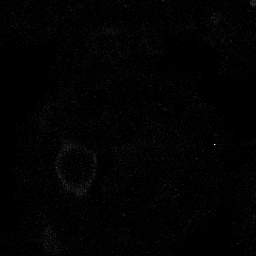

Supplement: Supplementary file 8 — Source data Fig. 5 [file 44319_2025_526_MOESM8_ESM.zip › Figure 5/5E/Nrf1cKO/merge_Nrf1cKO_Series002.tif]

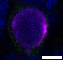

Supplement: Supplementary file 8 — Source data Fig. 5 [file 44319_2025_526_MOESM8_ESM.zip › Figure 5/5E/Nrf1cKO/merge_crop_Nrf1cKO_Series002.png]

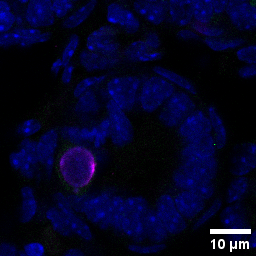

Supplement: Supplementary file 8 — Source data Fig. 5 [file 44319_2025_526_MOESM8_ESM.zip › Figure 5/5E/Nrf1cKO/merge_Nrf1cKO_Series002.png]

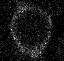

Supplement: Supplementary file 8 — Source data Fig. 5 [file 44319_2025_526_MOESM8_ESM.zip › Figure 5/5E/Nrf1cKO/merge_crop_Nrf1cKO_Series002.tif]

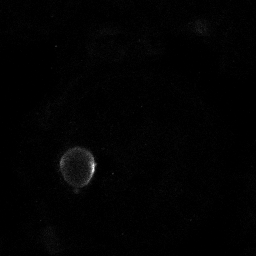

Supplement: Supplementary file 8 — Source data Fig. 5 [file 44319_2025_526_MOESM8_ESM.zip › Figure 5/5E/Nrf1cKO/TRA98_Nrf1cKO_Series002.tif]

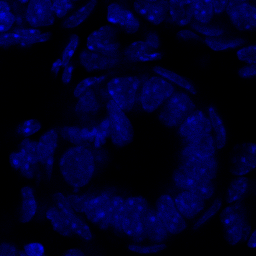

Supplement: Supplementary file 8 — Source data Fig. 5 [file 44319_2025_526_MOESM8_ESM.zip › Figure 5/5E/Nrf1cKO/DAPI_Nrf1cKO_Series002.png]

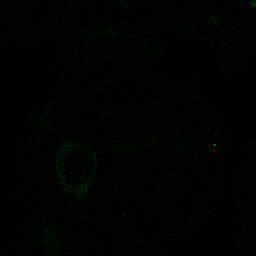

Supplement: Supplementary file 8 — Source data Fig. 5 [file 44319_2025_526_MOESM8_ESM.zip › Figure 5/5E/Nrf1cKO/L1ORF1_Nrf1cKO_Series002.png]

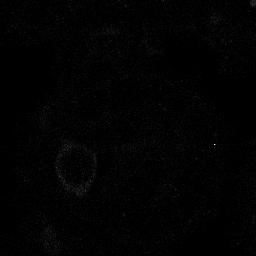

Supplement: Supplementary file 8 — Source data Fig. 5 [file 44319_2025_526_MOESM8_ESM.zip › Figure 5/5E/Nrf1cKO/L1ORF1_Nrf1cKO_Series002.tif]

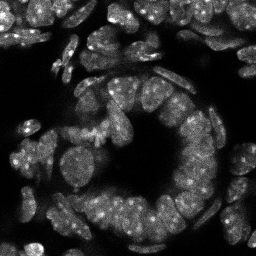

Supplement: Supplementary file 8 — Source data Fig. 5 [file 44319_2025_526_MOESM8_ESM.zip › Figure 5/5E/Nrf1cKO/DAPI_Nrf1cKO_Series002.tif]

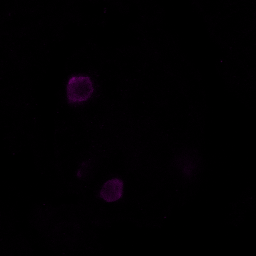

Supplement: Supplementary file 8 — Source data Fig. 5 [file 44319_2025_526_MOESM8_ESM.zip › Figure 5/5E/WT/TRA98_WT_Series008.png]

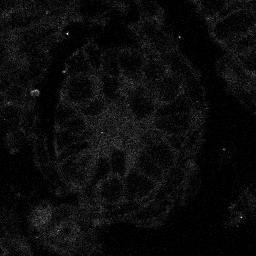

Supplement: Supplementary file 8 — Source data Fig. 5 [file 44319_2025_526_MOESM8_ESM.zip › Figure 5/5E/WT/merge_WT_Series008.tif]

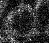

Supplement: Supplementary file 8 — Source data Fig. 5 [file 44319_2025_526_MOESM8_ESM.zip › Figure 5/5E/WT/merge_crop_WT_Series008.tif]

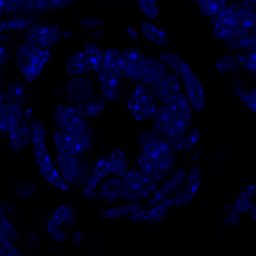

Supplement: Supplementary file 8 — Source data Fig. 5 [file 44319_2025_526_MOESM8_ESM.zip › Figure 5/5E/WT/DAPI_WT_Series008.png]

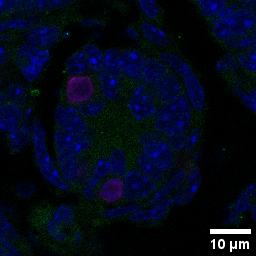

Supplement: Supplementary file 8 — Source data Fig. 5 [file 44319_2025_526_MOESM8_ESM.zip › Figure 5/5E/WT/merge_WT_Series008.png]

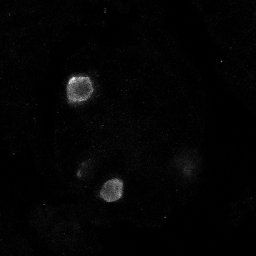

Supplement: Supplementary file 8 — Source data Fig. 5 [file 44319_2025_526_MOESM8_ESM.zip › Figure 5/5E/WT/TRA98_WT_Series008.tif]

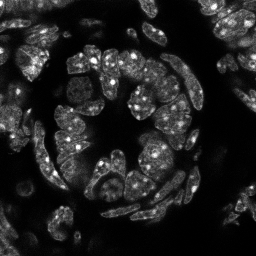

Supplement: Supplementary file 8 — Source data Fig. 5 [file 44319_2025_526_MOESM8_ESM.zip › Figure 5/5E/WT/DAPI_WT_Series008.tif]

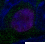

Supplement: Supplementary file 8 — Source data Fig. 5 [file 44319_2025_526_MOESM8_ESM.zip › Figure 5/5E/WT/merge_crop_WT_Series008.png]

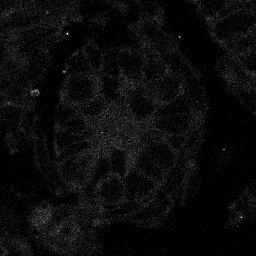

Supplement: Supplementary file 8 — Source data Fig. 5 [file 44319_2025_526_MOESM8_ESM.zip › Figure 5/5E/WT/L1ORF1p_WT_Series008.tif]

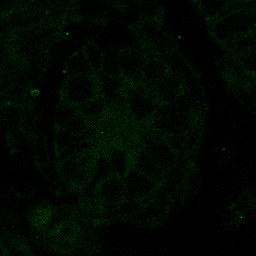

Supplement: Supplementary file 8 — Source data Fig. 5 [file 44319_2025_526_MOESM8_ESM.zip › Figure 5/5E/WT/L1ORF1p_WT_Series008.png]

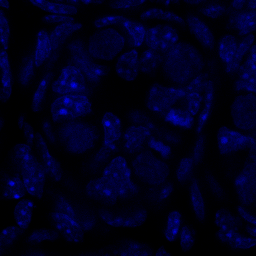

Supplement: Supplementary file 8 — Source data Fig. 5 [file 44319_2025_526_MOESM8_ESM.zip › Figure 5/5E/Dnmt3C_KO/DAPI_Dnmt3CKO_Series005.png]

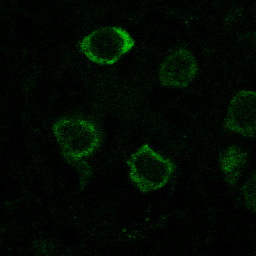

Supplement: Supplementary file 8 — Source data Fig. 5 [file 44319_2025_526_MOESM8_ESM.zip › Figure 5/5E/Dnmt3C_KO/L1ORF1p_Dnmt3CKO_Series005.png]

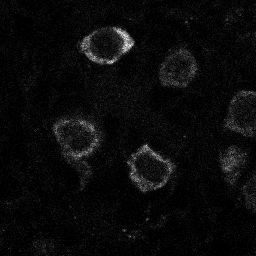

Supplement: Supplementary file 8 — Source data Fig. 5 [file 44319_2025_526_MOESM8_ESM.zip › Figure 5/5E/Dnmt3C_KO/merge_Dnmt3CKO_Series005.tif]

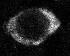

Supplement: Supplementary file 8 — Source data Fig. 5 [file 44319_2025_526_MOESM8_ESM.zip › Figure 5/5E/Dnmt3C_KO/merge_crop_Dnmt3CKO_Series005.tif]

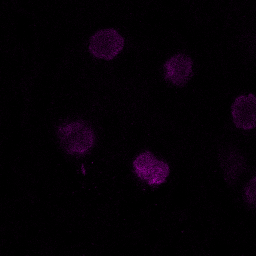

Supplement: Supplementary file 8 — Source data Fig. 5 [file 44319_2025_526_MOESM8_ESM.zip › Figure 5/5E/Dnmt3C_KO/TRA98_Dnmt3CKO_Series005.png]

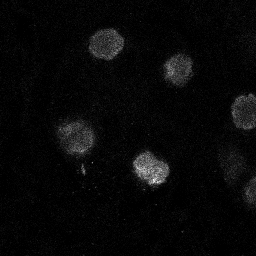

Supplement: Supplementary file 8 — Source data Fig. 5 [file 44319_2025_526_MOESM8_ESM.zip › Figure 5/5E/Dnmt3C_KO/TRA98_Dnmt3CKO_Series005.tif]

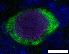

Supplement: Supplementary file 8 — Source data Fig. 5 [file 44319_2025_526_MOESM8_ESM.zip › Figure 5/5E/Dnmt3C_KO/merge_crop_Dnmt3CKO_Series005.png]

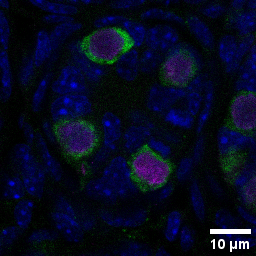

Supplement: Supplementary file 8 — Source data Fig. 5 [file 44319_2025_526_MOESM8_ESM.zip › Figure 5/5E/Dnmt3C_KO/merge_Dnmt3CKO_Series005.png]

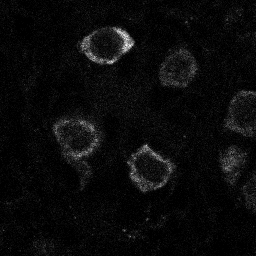

Supplement: Supplementary file 8 — Source data Fig. 5 [file 44319_2025_526_MOESM8_ESM.zip › Figure 5/5E/Dnmt3C_KO/L1ORF1p_Dnmt3CKO_Series005.tif]

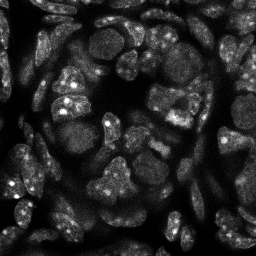

Supplement: Supplementary file 8 — Source data Fig. 5 [file 44319_2025_526_MOESM8_ESM.zip › Figure 5/5E/Dnmt3C_KO/DAPI_Dnmt3CKO_Series005.tif]

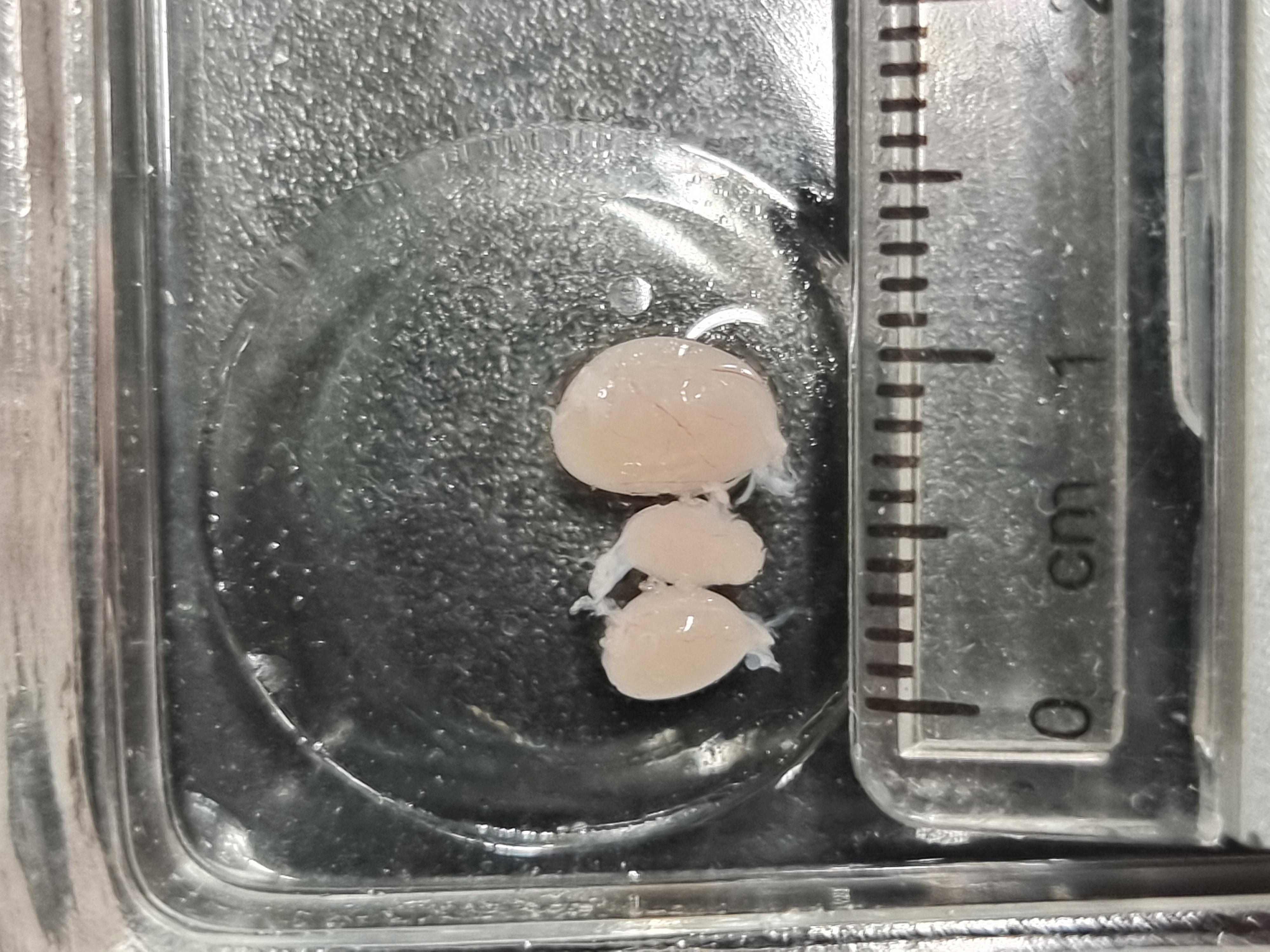

Supplement: Supplementary file 9 — Appendix Figure S8 Source Data [file 44319_2025_526_MOESM9_ESM.jpg]
