# Supplementary material for: DNA methylation at retrotransposons protects the germline by preventing NRF1-mediated activation
Source: EMBO Rep. 2025 Aug 4;26(17):4312–39. doi: 10.1038/s44319-025-00526-1 (PMC12420836; doi:10.1038/s44319-025-00526-1)
Supplement: Supplementary file 10 — Expanded View Figures [file 44319_2025_526_MOESM10_ESM.pdf]

Expanded View Figures

Figure EV1. Related to Fig. 1.

(**A**) Scheme showing percentage of mice testes cells with different age classified into developmental stages of Spg, Spc, spermatids and somatic cells. Meiosis I with Leptotene (L), Zygotene (Z), Pachytene (P) and Diplotene stages as well as Meiosis II are depicted in Spc. This image is modified from (Ernst et al, 2019). (**B**) Scheme showing the DNA methylation dynamics of wild-type and *Dnmt3C*$^{KO/KO}$ mouse germ cells across spermatogenesis. (**C**) Representative immunostaining from biological duplicates of LINE1-ORF1p and TRA98 (germ cell marker) on cryosections of wild-type (WT) and *Dnmt3C*$^{KO/KO}$ testes at specific developmental time points centered on one representative germ cell nucleus. Zoom10, Scale bar, 5 µm. (**D**) Box plots displaying the mean intensity of LINE1-ORF1p quantification from three nuclei as in (**C**). Error bars represent 1.5 times the interquartile range (IQR) above and below the median, where IQR is defined as the difference between the third and first quartiles.

 © The Author(s)

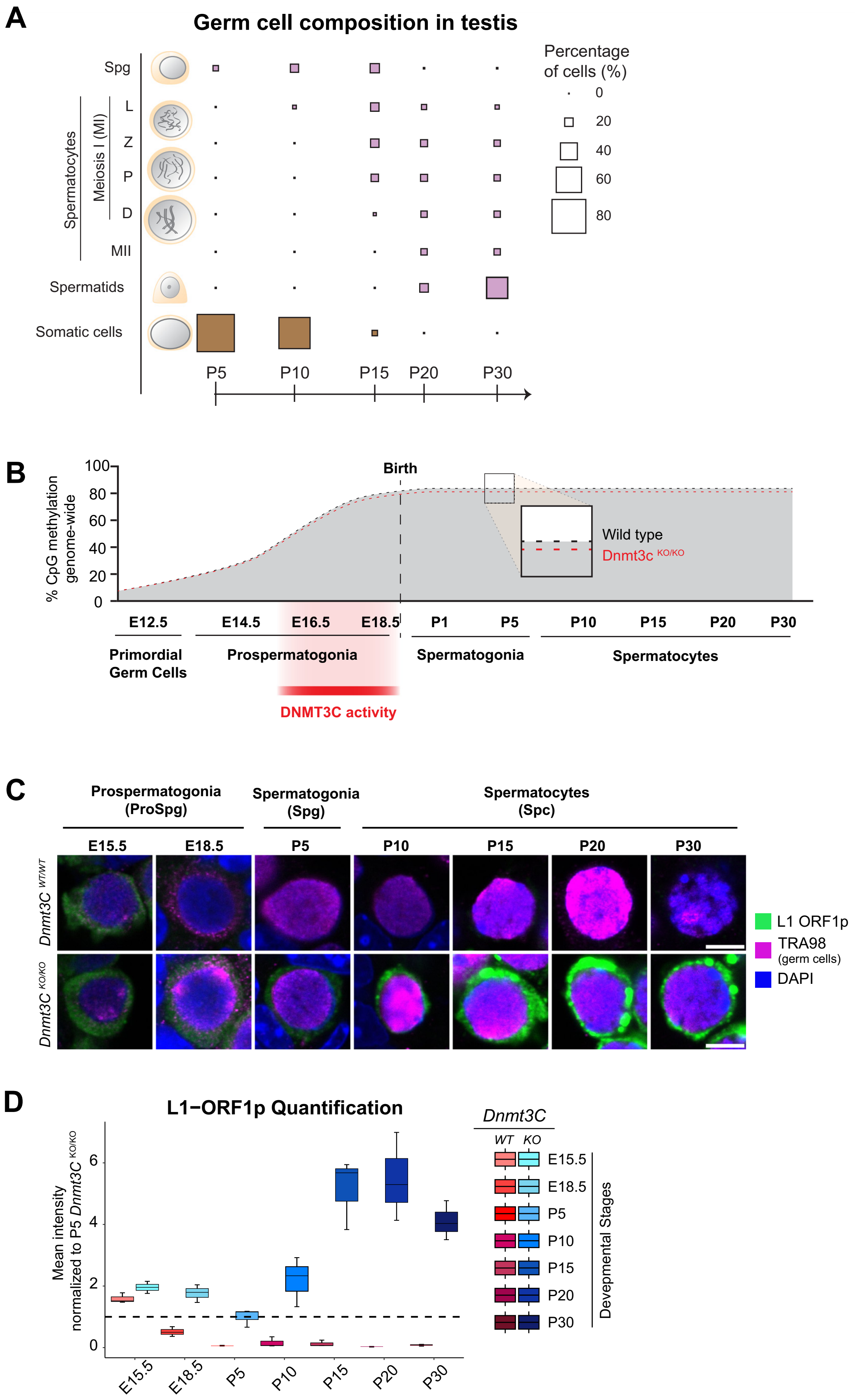
A
Germ cell composition in testis
Spg
Spermatocytes
Meiosis I (MI)
L
Z
P
D
MII
Spermatids
Somatic cells
P5
P10
P15
P20
P30
Percentage of cells (%)
0
20
40
60
80
B
% CpG methylation genome-wide
100
80
60
40
20
0
Birth
Wild type
Dnmt3c KO/KO
E12.5
E14.5
E16.5
E18.5
P1
P5
P10
P15
P20
P30
Primordial Germ Cells
Prospermatogonia
Spermatogonia
Spermatocytes
DNMT3C activity
C
Prospermatogonia (ProSpg)
Spermatogonia (Spg)
Spermatocytes (Spc)
E15.5
E18.5
P5
P10
P15
P20
P30
Dnmt3C WT/WT
Dnmt3C KO/KO
L1 ORF1p
TRA98 (germ cells)
DAPI
D
L1−ORF1p Quantification
Mean intensity normalized to P5 Dnmt3C KO/KO
6
4
2
0
E15.5
E18.5
P5
P10
P15
P20
P30
Dnmt3C
WT KO
Devepmental Stages

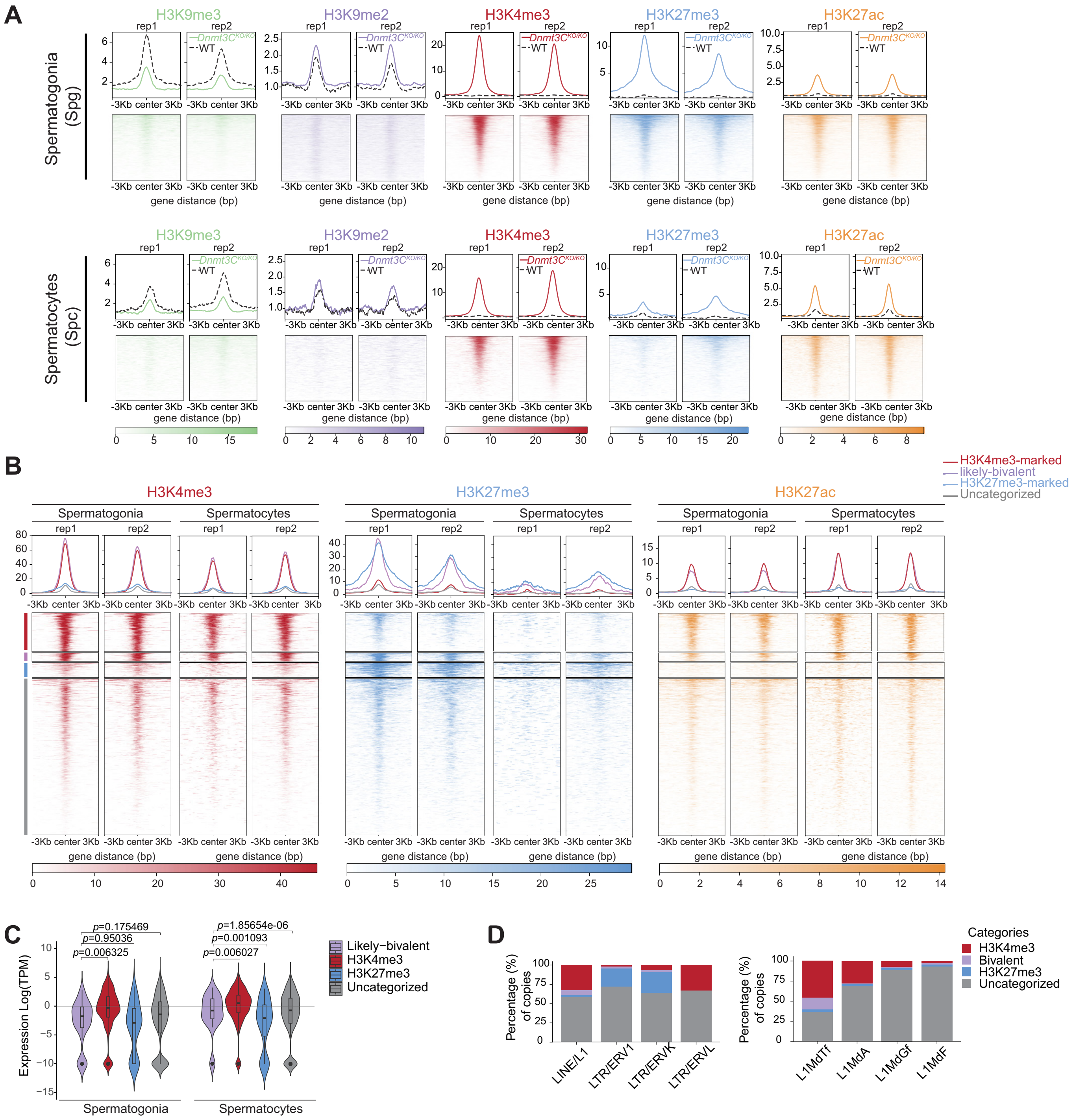

Figure EV2. Related to Fig. 2.

(**A**) Heatmaps displaying normalized coverage and metaplots showing mean enrichment of H3K4me3, H3K27me3, H3K9me2, H3K9me3 and H3K27ac CUT&Tag from uniquely mapped reads of two biological replicates (rep), centered on DNMT3C targets in sorted *Dnmt3C*$^{KO/KO}$ Spg and Spc. The corresponding mean enrichment in wild-type is illustrated as a dashed black line in the same metaplot. (**B**) Heatmaps showing normalized coverage and metaplots displaying mean enrichment of H3K4me3, H3K27me3 and H3K27ac CUT&Tag from uniquely mapped reads centered on likely bivalent, H3K4me3-marked, H3K27me3-marked and uncategorized DNMT3C targets in sorted *Dnmt3C*$^{KO/KO}$ Spg and Spc. (**C**) Violin plots with boxplots showing log(TPM) values from RNA sequencing of three biological replicates from TElocal of likely bivalent, H3K4me3-marked, H3K27me3-marked and uncategorized DNMT3C targets from uniquely mapped reads in *Dnmt3C*$^{KO/KO}$ Spg and Spc. The median and IQR range of the boxplot are defined as in Fig. 2C. *P* values were tested using an unpaired *t* test between likely bivalent clusters and other clusters of the same stage and genotype. (**D**) Column charts showing the percentage of TE classes and families classified as likely bivalent, H3K4me3-marked, H3K27me3-marked, uncategorized DNMT3C targets from uniquely mapped reads of two biological replicates.

 © The Author(s)

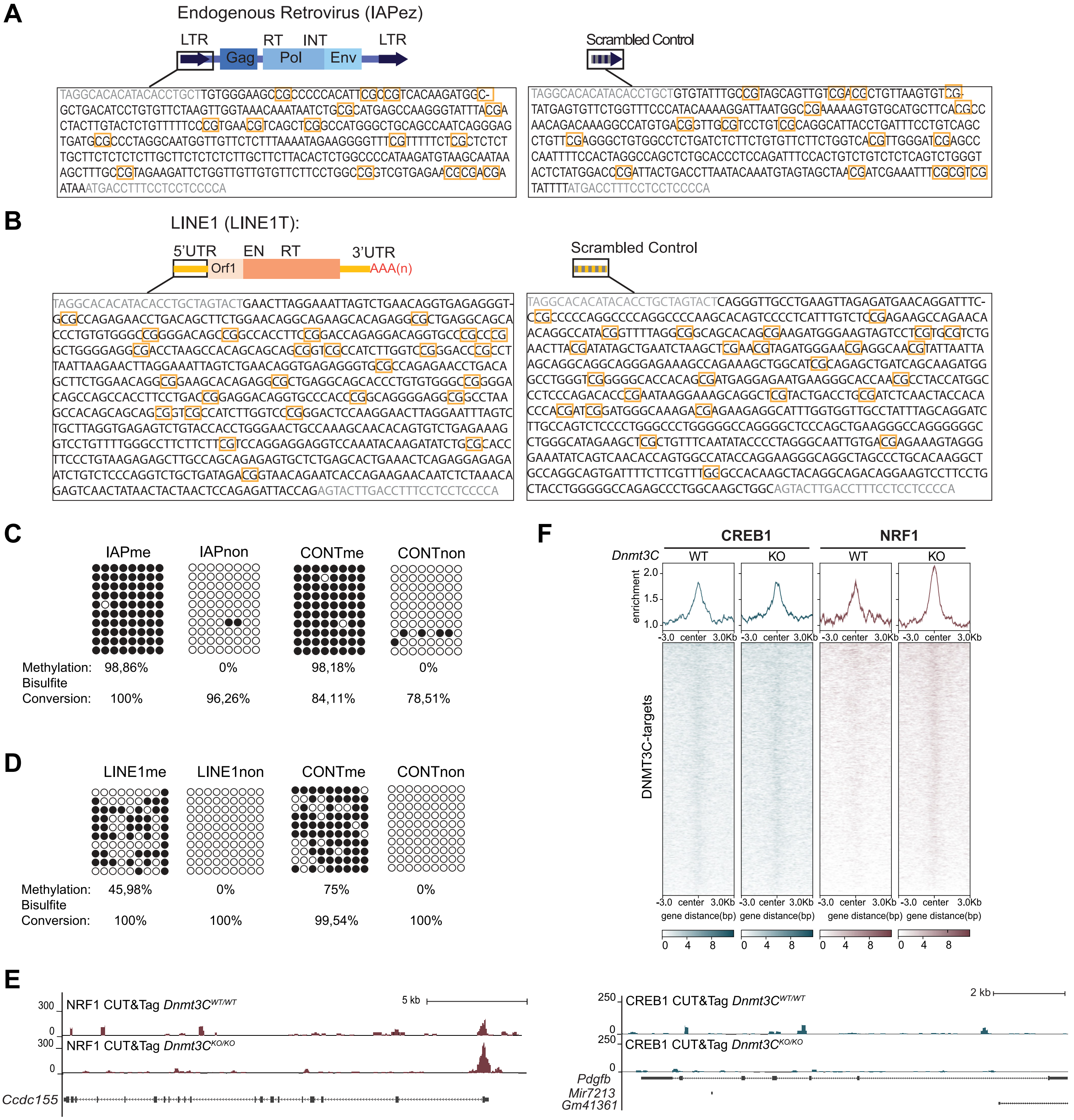

Figure EV3. Related to Fig. 3.

(**A**) Illustration of the bait sequence used from the IAP LTR1a promoter, and the corresponding scrambled control used in the DNA-pulldown experiment. CpG sites, which are identical between IAP LTR1a and the scrambled control, are highlighted with a yellow frame. The sequences corresponding to random primers used for amplification and biotinylation, are shown in gray. (**B**) as in (**A**) for the LINE1MdT promoter and its scrambled control. (**C**) Bisulfite cloning results for IAPme, IAPnon, CONTme, and CONTnon, represented as black/white circle diagrams, with black circles indicating methylated cytosines and white I indicating unmethylated. Each column is a CpG site, and each row represents a biological replicate (n=10). Average methylation percentages and bisulfite conversion rates are indicated at the bottom. (**D**) As in (**C**) for LINE1me, LINE1non, CONTme and CONTnon. (**E**) Representative track example of CREB1 and NRF1 CUT&Tag example at bona fide targets *Pdgfb* and *Ccdc155*, respectively. (**F**) Heatmaps displaying normalized coverage and metaplots showing mean enrichment of CREB1 and NRF1 at DNMT3C targets in wild-type and *Dnmt3C*$^{KO/KO}$ Spermatogonia (Spg) merged from two biological replicates.

© The Author(s)

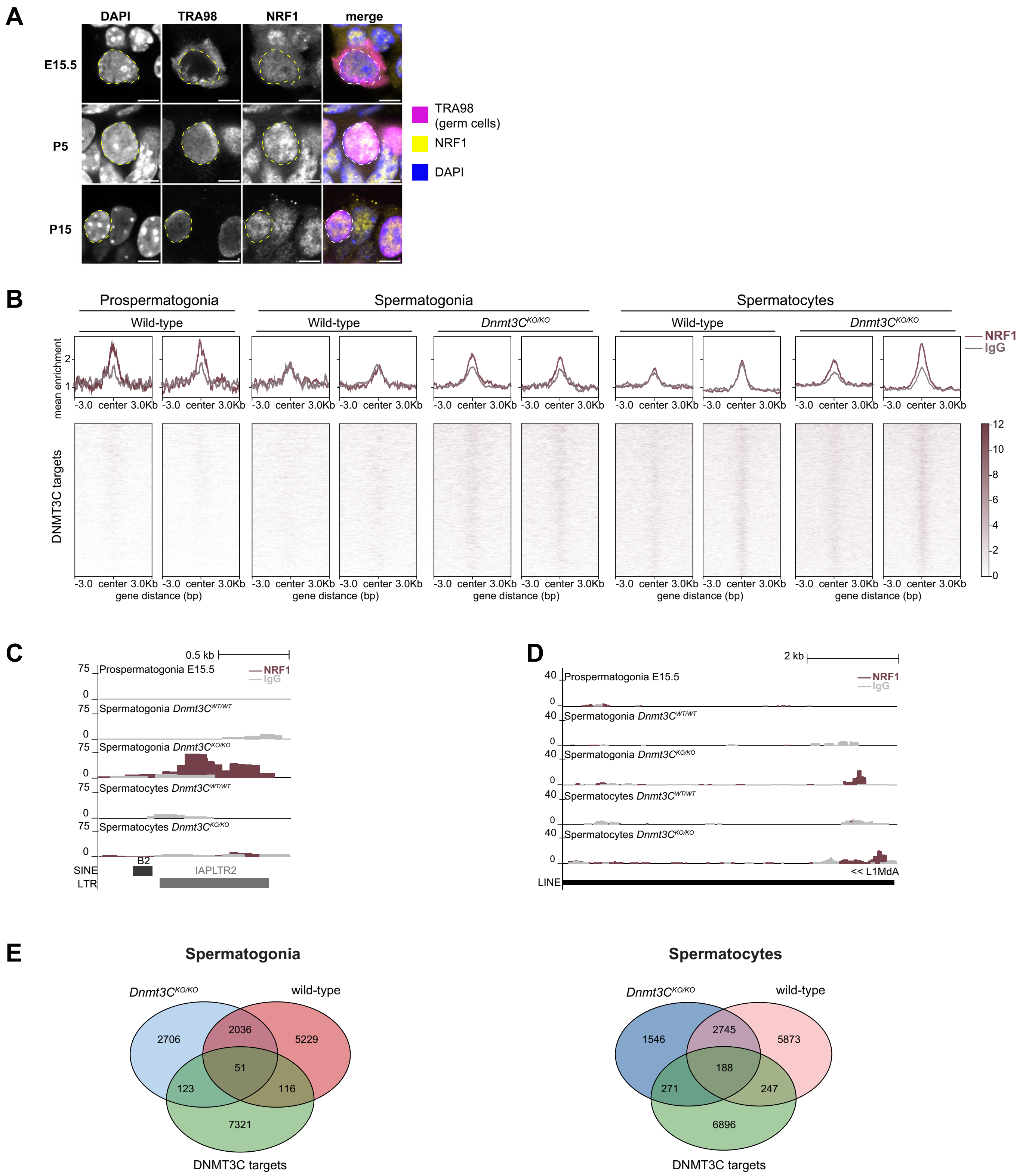
A
DAPI
TRA98
NRF1
merge
E15.5
P5
P15
TRA98 (germ cells)
NRF1
DAPI
B
Prospermatogonia
Spermatogonia
Spermatocytes
Wild-type
Dnmt3C KO/KO
NRF1
IgG
mean enrichment
DNMT3C targets
-3.0 center 3.0Kb
gene distance (bp)
C
0.5 kb
Prospermatogonia E15.5
Spermatogonia Dnmt3C WT/WT
Spermatogonia Dnmt3C KO/KO
Spermatocytes Dnmt3C WT/WT
Spermatocytes Dnmt3C KO/KO
SINE
LTR
B2
IAPLTR2
D
2 kb
LINE
<< L1MdA
E
Spermatogonia
Dnmt3C KO/KO
wild-type
2706
2036
5229
51
123
116
7321
DNMT3C targets
Spermatocytes
1546
2745
5873
188
271
247
6896

 © The Author(s)

Figure EV4. Related to Fig. 4.

(**A**) Immunostaining of testes cryosections at E15.5, P5 and P15 showing TRA98 (germ cell marker) and NRF1 in the nucleus from one biological replicate, which is illustrated by DAPI and highlighted with a dashed yellow line. Scale bar 5 µm. (**B**) Heatmaps displaying normalized coverage and metaplots showing mean enrichment of NRF1 at DNMT3C targets wild-type and *Dnmt3C*$^{KO/KO}$ Spg and Spc from two biological replicates. The corresponding mean enrichment of the IgG control is illustrated as a gray line in the same metaplot. (**C**) Representative track example of NRF1 CUT&Tag as in (**D**) at an IAPLTR2 (ERVK) copy located at chr11: 101,391,538–101,402,538. (**E**) Venn diagrams showing overlaps of NRF1 peaks with DNMT3C targets in wild-type and *Dnmt3C*$^{KO/KO}$ Spermatogonia and Spermatocytes.

© The Author(s)

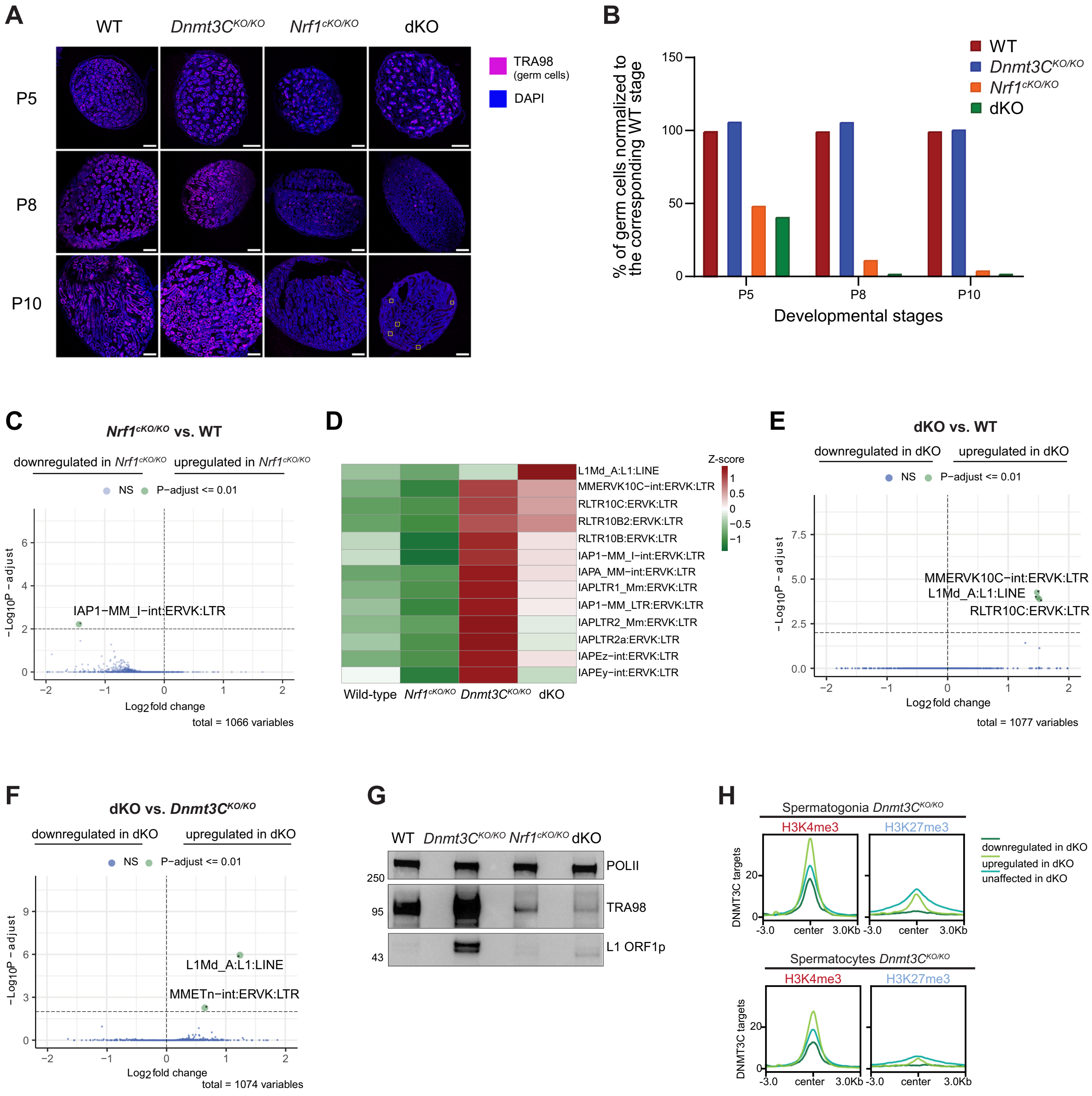

Figure EV5. Related to Fig. 5.

(**A**) Immunostaining of whole-testis cryosections showing TRA98 (germ cell marker) and DAPI at P5, P8 and P10 for wild-type, *Dnmt3C*$^{KO/KO}$, *Nrf1*$^{cKO/KO}$ and dKO (*Dnmt3C*$^{KO/KO}$, *Nrf1*$^{cKO/KO}$) from one biological replicate. Scale bar 200µm. TRA98-marked cells in P10 dKO are marked with yellow boxes for illustration. (**B**) Bar diagram showing germ cell percentage from the mutants in different stages according to panel (**A**). TRA98/DAPI counts generated by Fiji ImageJ automated counting from one stage were normalized to TRA98/DAPI counts in the corresponding WT stage from one biological replicate. (**C**) Volcano plot showing DESeq2 results with $\log_2$ FC and -$\log_{10}$p-values from Wald test comparing wild-type versus *Nrf1*$^{cKO/KO}$ from three biological replicates. TE names displayed following the pattern TEname:family:TEclass. (**D**) Heatmaps showing Z-score of TEtranscripts from three biological replicates from differentially expressed TE families in all mutants. TE names displayed following the pattern TEname:family:TEclass. (**E**) As in (**C**) for dKO vs. wild-type. (**F**) As in (**C**) for dKO vs. *Dnmt3C*$^{KO/KO}$. (**G**) Western blot analysis of POL II, TRA98 and LINE1-ORF1p on protein extracts from wild-type, *Dnmt3C*$^{KO/KO}$, *Nrf1*$^{cKO/KO}$ and dKO testes at P8 from one biological replicate. The blot was developed using femto chemistry. (**H**) Metaplots showing mean enrichment of H3K4me3 and H3K27me3 CUT&Tag from two biological replicates in wild-type and *Dnmt3C*$^{KO/KO}$ Spg and Spc, centered on DNMT3C targets that were upregulated, downregulated or unaffected in dKO compared to *Nrf1*$^{cKO/KO}$.

 © The Author(s)
